# Microtubule inhibition as a proposed mechanism for the anthelmintic effect of phytochemicals isolated from *Cicerbita alpina*

**DOI:** 10.1038/s41598-024-73958-9

**Published:** 2025-02-03

**Authors:** Mark James Horgan, Ines Sigg, Ioanna Poulopoulou, Francisco J. Rodriguez-Mejias, Eva Albertini, Pietro Fusani, Florian Fischer, Eftychia Martinidou, Daniela Schuster, Stefan Martens, Pidder Jansen Dürr, Matthias Gauly, Hermann Stuppner, Alexander Weiss, Veronika Temml, Bianka Siewert

**Affiliations:** 1 Institute of Pharmacy/Pharmacognosy, Center for Chemistry and Biomedicine, University of Innsbruck, 6020 Innsbruck, Austria; 2 Research Institute for Biomedical Aging Research, University of Innsbruck, 6020 Innsbruck, Austria; 3 Faculty of Agricultural, Environmental and Food Sciences, Free University of Bolzano, Piazza Università 5, 39100 Bolzano, Italy; 4 Present Address: Department of Animal Science, Agricultural University of Athens, Iera Odos 75, 118 55 Athens, Greece; 5 Centro Di Ricerca Foreste E Legno, Consiglio Per La Ricerca in Agricoltura E L’analisi Dell’economia Agraria, Piazza Nicolini 6 Loc. Villazzano, 38123 Trento, Italy; 6 Institute of Pharmacy, Department of Pharmaceutical and Medicinal Chemistry and Research and Innovation Center for Novel Therapies and Regenerative Medicine, Paracelsus Medical University Salzburg, Strubergasse 21, 5020 Salzburg, Austria; 7 Research and Innovation Center, Edmund Mach Foundation, Via E. Mach 1 38098 - San Michele All’Adige, Trento, Italy; 8 Present Address: Allelopathy Group, Department of Organic Chemistry, School of Sciences, Institute of Biomolecules (INBIO), University of Cadiz, C/Republica Saharaoui 7, 11510 Puerto Real, Cadiz, Spain; 9 Center of Molecular Biosciences Innsbruck (CMBI), University of Innsbruck, Innsbruck, Austria

**Keywords:** Target identification, Infection, Drug development

## Abstract

The alpine plant *Cicerbita alpina* (L.) Wallr., when grown as a sprout, is known as a bitter-tasting culinary delicacy. Recently it has also been reported to have anthelmintic activity, prompting further investigation into its mechanism of action. Liquid–liquid fractions were prepared from a methanolic extract of the aerial parts and were submitted in parallel to embryo development (ED), worm motility (WMT), and cytotoxicity assays for anthelmintic and toxicity evaluations. The anthelminthic assays revealed the more polar fractions to be most active against *Ascaridia galli* embryos (BuOH | 68% ED | c = 500 µg/ml and EtOAc | 65% ED | c = 500 µg/ml) and *Caenorhabditis elegans* adult worms (BuOH | 49% WMT | c = 150 µg/ml and EtOAc | 74% WMT | c = 150 µg/ml) suggesting the fraction’s constituents possess dual anthelmintic activity against multiple life-cycle stages (i.e., eggs, worms) of helminths. Additionally, the BuOH fraction was non-cytotoxic to human cell-lines. Subsequent FCC and SEC derived subfractions were submitted to the anthelmintic assay workflow and the enriched subfractions B1 and E3.8, phytochemically assigned as 11-β,13-dihydrolactucin and luteolin, demonstrated bioactivity against the embryo phenotype (B1 | 58% ED | c = 1.8 µM and E3.8 | 46% ED | c = 1.7 µM) within range of the flubendazole control. Furthermore, luteolin was found to inhibit *C. elegans* egg hatching (luteolin | 65% EH | c = 10 µM | t = 10 h) within the range of the control albendazole. Both identified anthelmintic phytochemicals were found to affect tubulin polymerisation at a concentration of c = 50 µM. Together with in silico virtual screening studies, these results suggest microtubule stabilisation as a possible anthelmintic target and mechanism of action. This work effectively advocates the consideration of *C. alpina* extracts and fractions for the development of herbal therapeutics against parasitic helminths.

## Introduction

Helminth worms are common parasitic nematodes that afflict the gastrointestinal (GI) tract of their host and remain a significant global health threat. It is estimated that over a quarter of the human population (> 1.5 billion), mostly in developing countries, are infected by soil-transmitted helminths, representing a major category of neglected tropical diseases (NTD) ^1,2^. Furthermore, helminth parasites are an economic burden to livestock farming worldwide and threaten international food security ^3,4^. Classically, helminth infections are combated via the mass administration of synthetic anthelmintic drugs ^2^. However, the sustainability of this approach is continually threatened by the increased prevalence of anthelmintic drug resistance to treatments in both human and veterinary medicine ^5,6^. The alarming limitation of this control strategy thus requires novel solutions to find alternative or complementary treatments. One possible solution is the use of plant extracts or their derived natural products for worm control. Historically, natural products have provided important lead scaffolds toward the development of clinically relevant antiparasitic medications, including but not limited to, santonin, artemisinin, and ivermectin ^7–9^. Therefore, natural products warrant further investigation and should be prioritised as screening candidates in the effort to discover novel anthelmintic treatments.

However, preclinical anthelmintic drug discovery faces notable challenges and drawbacks during in vitro screening investigations. Firstly, procuring a host infected with parasites in the wild can often be seasonal and sporadic, thus making it difficult to collect adequate amounts of biomaterial for testing. Second, the culturing and life-cycle stage maintenance of parasitic worms in vitro without the host is extremely challenging and cost-intensive ^10,11^. To circumvent these limitations, the closely related free-living *Caenorhabditis elegans* is often used as a model organism for anthelmintic phenotypic screens. This nematode is favoured due to its ease of cultivation, high fecundity, and low maintainance cost ^11,12^. Moreover, recent worm motility phenotype screening campaigns employing the WMicrotracker instrument have demonstrated the combined utility of this model organism and high-throughput platform in natural products anthelminthic research ^13,14^.

Alpine cultivated plants, predominantly members of the Asteraceae family, have recently demonstrated anthelmintic activities in vitro and could be considered as alternative natural sources for deworming purposes ^15^. Results showed that among ten plants screened, the methanolic extract of *Cicerbita alpina* (L.) Wallr. was found to be the most active at inhibiting in vitro embryo development of *Ascaridia galli* eggs ^15^. Nevertheless, this previous study was limited to one life cycle stage (i.e., eggs) and therefore limited the evaluation of the true potential of this extract, as established anthelmintic drugs often target helminths at multiple developmental stages to effectively eradicate the parasitic burden. Furthermore, to establish an herbal extract as a natural dewormer, standardised extracts and monographs need to be developed to meet the regulatory standards. Therefore, knowledge regarding the active principle(s) is required.

Thus, the objective of this study is twofold: (1) exploration whether the *C. alpina* extract and its subsequent fractions possess dual anthelmintic activity by targeting multiple helminth life cycle stages (i.e., worms, eggs); (2) annotation of the active principle(s) to provide the basis for future quality control. To achieve these goals, we also worked on the implementation of a fast and orthogonal screening method based on the cultivable helminth *C. elegans*.

## Material and methods

### Plant material, extraction, fractionation, and isolation

Cultivation of *C. alpina* was carried out at the Centro di ricerca Foreste e Legno in Trento, Italy, starting with seeds collected from the wild. Seed collection was carried out in accordance with local laws and with the appropriate permits. Cultivation was carried out according to national and local (Autonomous Province of Trento) laws. The material has been identified by Pietro Fusani and voucher specimens (#CA20190709A and #CA20190709B) are deposited in the herbarium of the CREA Research Centre for Forestry and Wood (Consiglio per la ricerca in agricoltura e l’analisi dell’economia agraria, Centro di ricerca Foreste e Legno, piazza Nicolini 6 loc. Villazzano, 38,123 Trento, Italia).

Dry ground aerial parts of *C. alpina* (m = 498.4 g) were first defatted with petroleum ether (V = 2.5 L) via multiple sonication steps (t = 15 min, n = 4). Between cycles, the material was filtered through a large Buckner funnel to collect the solvent. A final sonication (V = 1 L, t = 15 min, n = 1) and maceration (t = 48 h) was performed, and after decanting, the plant material was left to dry under an air stream for 48 h. The above sonication extraction was repeated on the dry and defatted plant material using methanol. The solvent was evaporated and the extract concentrated *in vacuo*, yielding a viscous crude methanol (MeOH) extract which was freeze-dried (m = 23.7 g, η = 4.8 % d.w.). To prepare fractions, part of the methanol extract (Ca_MeOH) obtained (m = 21.0 g) was submitted to a liquid–liquid separation procedure using solvents by order of increasing polarity (petroleum ether (PE), dichloromethane (DCM), ethyl acetate (EtOAc), and butanol (BuOH)) in a glass separatory funnel. This fractionation yielded a petroleum ether (PE) fraction (η = 2.1 g), a DCM fraction (η = 6.0 g), an EtOAc fraction (η = 1.7 g), and a BuOH fraction (η = 3.4 g).

Eight subfractions of the BuOH fraction (m = 2.66 g) were prepared via silica-gel flash column chromatography (FCC) using CHCl_3_:MeOH (9:1) yielding subfractions (**B1**–**B3**) and (8:2) yielding (**B4**–**B7**) and a final wash with MeOH (**B8**); with the following yields **B1** (η = 37.3 mg), **B2** (η = 9.6 mg), **B3** (η = 11.7 mg), **B4** (η = 25.5 mg), **B5** (η = 111.8 mg), **B6** (η = 189.6 mg), **B7** (η = 53.0 mg), and **B8** (η = 2.18 g).

Thirteen subfractions of the EtOAc fraction (m = 1.20 g) were also prepared via silica-gel FCC using CHCl_3_:MeOH (9:1) yielding subfractions (**E1**–**E4**) and (8:2) (**E5**–**E10**) and washed with MeOH (**E11–E13**); with the following yields **E1** (η = 249.4 mg), **E2** (η = 84.4 mg), **E3** (η = 66.0 mg), **E4** (η = 31.5 mg), **E5** (η = 29.4 mg) **E6** (η = 10.7 mg) **E7** (η = 139.0 mg), **E8** (η = 63.7 mg), **E9** (η = 95.4 mg), **E10** (η = 53.6 mg), **E11** (η = 123.5 mg), **E12** (η = 29.3 mg) and **E13** (η = 72.5 mg). A sample of the fraction **E3** (m = 56.0 mg) was further fractionated via size exclusion chromatography (SEC) yielding samples **E3.1**–**E3.8**.

#### Targeted isolation of 11β, 13-dihydrolactucin derivative

Air-dried leaves and shoots of *C. alpina* (m = 2.2 kg) were ground. Portions (m = 500 g, n = 4) of the total amount were sequentially extracted with DCM (V = 1.2 L, n = 4) and MeOH (V = 0.7 L, n = 4) each. The combined DCM extract was concentrated under vacuum to yield a crude extract (η = 165.7 g) which was further fractionated by FCC. The method employed was carried out with a Reveleris® X2 equipment (Buchi, Flawil, Swiss), using a FlashPure EcoFlex Silica cartridge (220 g, irregular particle shape, 40–63 µm particle size, and 55–75 Å pore size). After an equilibration time (t = 5 min) with pure hexane, a gradient of hexane–EtOAc (from 95:5 to 60:40) followed (t = 59 min) resulting in 37 subfractions. A portion of subfraction #7 (η = 12.9 g in total) was subjected to a SEC (Sephadex LH20, h = 870 mm, Ø = 25 mm) using an isocratic mixture of DCM and acetone (85:15) as mobile phase. This separation generated 23 subfractions. Fraction #7.17 (η = 126.7 mg) was separated by preparative HPLC (Agilent Technologies, Santa Clara, CA, United States) using a Synergi Polar-RP® column (Phenomenex, Aschaffenburg, Germany, l = 250 mm, Ø = 21.2 mm, 4 µm, 80 Å). A THF solution of 15 mg/mL was filtered through cotton wool and injected for separation. A gradient (90:10 to 55:45) of water acidified with formic acid (0.9%) and acetic acid (0.1%) and ACN was employed at a flow rate of *Q* = 0.6 mL/min, the column oven was set to a temperature of T = 30 °C, and the injection volume was adjusted to V = 50 µL. 11β, 13-dihydrolactucin (η = 6.4 mg, 5% w/w_fraction_, 0.003% w/w_DCM-Extract_) was obtained as a white powder. See SI 2.2.2 for chemical characterisation.

### Phytochemical analysis

#### High performance liquid chromatography (HPLC) and mass spectrometry (MS)

Samples of the fractions and subfractions were dissolved in MeOH or DMSO (c = 4 mg/mL) and transferred to ambered coloured HPLC vials via cotton wool filtration. The samples, including the blank, were submitted to HPLC–PDA and HPLC–DAD-MS measurements. For HPLC analysis, a Shimadzu HPLC–UFLC XR system with a photodiode-array detector (PDA) was utilised (Shimadzu, Kyoto, Japan). A Luna column (Phenomex, Emeryville, CA, USA, 3u, C8 (2), 100 (Å), Ø = 4.6 mm, l = 100 mm) was employed with a pre-column (C18, 4 × 3 mm). The mobile phase consisted of water (Phase A) acidified with formic acid (0.9%) and acetic acid (0.1%) and MeOH (Phase B). The column temperature was set to T = 35 °C, a flow rate of *Q* = 0.6 mL/min was set and an injection volume of V = 10 μL applied. With a runtime of t = 52 min, the employed gradient system started with 15% B at 0.01 min, reaching 27.5% B after 25 min and increasing to 90% B at 47 min, where it was set on hold for 5 min. The PDA detection wavelength (λ = 254 nm) was selected for spectrophotometric analysis.

The HPLC–DAD-MS measurements were performed with the same gradient and solvent system using an Agilent 1260 Infinity instrument (Agilent technologies, Santa Clara, CA, USA) including a quaternary pump, vial sampler, column thermostat, UV detector, and coupled to an amaZon SL quadrupole ion trap mass spectrometer (Bruker, Billerica, MA, USA).

The MS parameters were set as follows: API-ES spray chamber, drying gas (T = 320 °C, *Q* = 12 L/min), nebuliser pressure = 25 psi, scanning range m/z = 200–1200, capillary voltage = 4.5 kV. To determine mass, ionisation was carried out by electrospray ionisation (ESI) in both positive and negative modes and a quadrupole ion trap was used for detection. Analysis of the obtained chromatograms was performed by exploring the UV–Vis spectra as well as the MS traces of the major peaks in both positive and negative mode. Where possible comparisons were made to reference substances and literature values. Chromatograms were processed and analysed in OriginPro, 2020 (OriginLab, Northampton, MA, USA).

NMR spectra were recorded on two spectrometers from Bruker, an Avance II 600 spectrometer operating at 600 MHz (^1^H) and an Avance III HD spectrometer operating at 400 MHz (^1^H) (Bruker Corporation, Billerica, USA).

### Anthelmintic activity evaluation experiments

#### Anthelmintic worm motility assay (WMT) with *C. elegans.*

The wild-type *C. elegans* N2 Bristol strain was obtained from the Caenorhabditis Genetics Center (CGC, University of Minnesota, MN, USA). To prepare for anthelmintic screening, the worms were grown on Nematode Growth Medium (NGM) in Petri dishes seeded with a lawn of *Escherichia coli* OP50-1 bacteria ^16^. The bacteria culture and NGM were prepared under sterile conditions according to established formulations ^17^. Synchronized populations of adult worms were obtained by the previously described alkaline bleaching method ^18,19^ with some modifications. Worm culturing and synchronisation procedures are outlined in detail in the supplementary part (SI, Sect. 1.1).

The anthelminthic screening assay was performed in a 96-well microplate (flat-bottom, Nunclon®, Thermo Fisher Scientific Inc., Waltham, MA, USA). Synchronised adult worms were collected in S-medium (V = 8–12 mL) with the suspension volume adjusted until the average number of worms was calculated  (around 35–50 worms/per 100 µL, V ~ 10 mL). A liquid culture was prepared from the worm suspension by adding OP50-1/S-medium (c_stock_ = 200 mg/mL, V = 184 µL) in (OD_600_= 0.6).

Then, the final liquid culture suspension (V =  ~ 10 mL) was distributed across the 96-well microplate (V = 100 µL per well). The worms/plate were left to shake gently on a wave shaker (t = 20 min, frequency = ~ 10 rpm, waving motion) and after, the microplate was submitted to the WMicrotracker (Phylumtech, Sunchales, Argentina) (t = 30 min) to measure the basal worm motility. Each extract, fraction, or pure compound stock solution was prepared in DMSO and further diluted with S-medium. Subsequently, each diluted test solution or controls were added to the desired wells (V = 20 µL each) reaching the final concentrations of c = 150, 100, 50 µg/mL, or -where insoluble- of c = 75, 50, 25 µg/mL with DMSO (1%). Ivermectin (abcr GmbH, Karlsruhe, Germany) (c_final_ = 0.1 µM, in 1% DMSO) was used as a positive control and the vehicle negative control was DMSO (1%, v/v). After shaking (t = 2 min, frequency = ~ 10 rpm, waving motion), the 96-well microplate was placed into the WMicrotracker and incubated (up to t = 12 h, T = 24 °C). Worm motility (WMT) within each well was measured every thirty minutes and motility recorded as activity counts per defined time intervals by the WMicrotracker. The relative WMT anthelminthic activity of the test samples was estimated by normalising the values to their respective basal activity value and then expressed as a percentage of the DMSO (1%) control with average worm motility shown over four hours.

#### Anthelmintic egg hatch assay (EHA) with *C. elegans*

Worms were chunked from a stock plate containing a mixed population of N2 Bristol worms and transferred to a freshly prepared OP50-1 seeded NGM plate and left to incubate until the development of large numbers of gravid adults and eggs were visibly laid on the plate. Next, eggs were harvested and isolated by bleaching (SI, Sect. 1.2). The eggs were collected in S-medium and the volume was adjusted until an average of ~ 150 embryos/100 µL was counted. Thereafter, the egg/S-medium suspension (V = 100 µL) was distributed to the wells of a 96-well microplate (flat-bottom, Nunclon®, Thermo Fisher Scientific Inc.,  Waltham, MA, USA) and the positive control albendazole (TCI Chemicals, Zwijndrecht, Belgium) ( c_final_ = 10 µM) or test sample was added (V = 20 µL). Next, the plate was gently shaken (t = 2 min, frequency = ~ 10 rpm, waving motion) and then placed into the WMicrotracker and incubated (t = 24 h, T = 24 °C). Egg hatching was monitored by the mean number of beam interruptions during the experiment.

#### Anthelmintic embryo development assay with *A. galli*

The isolation of *A. galli* eggs was performed as previously described ^15^ with further detail outlined (SI Sect. 1.2). Starting from the day of egg isolation (d = 0) onwards, the embryonic development of eggs (*in ovo* larval development) was evaluated for each petri dish (n = 27, samples tested in duplicate) by examining the morphological characteristics of embryos within the eggs. Each petri dish was loaded with eggs (n = 250) and the respective test solutions (c = 0.5 mg/ml, 0.5% formalin, 1% DMSO).The embryonic development was evaluated every second day until day 28 . Untreated petri dishes as negative controls included petri dishes with medium containing 0.5% formalin and 1% DMSO. Flubendazole (c = 1.6 µM) was used as a positive control. At every measurement, eggs (n = 20) were selected randomly, and their embryonic developmental stage was evaluated. The examined eggs were classified as either undeveloped or into different development stages ^15,20^. Throughout the experimental period 12,240 eggs were evaluated in total. The percentage of the eggs that corresponds to each development class (e.g., undeveloped, early development etc.) was calculated according to the following equation:

$$\text{Calculate Developed Embryos }\left(\text{\%ED}\right)= \frac{\#{Eggs}_{total}-\#{Eggs}_{undeveloped}}{\#{Eggs}_{total}}$$

### Cytotoxicity assay on malignant human X cells

Cells of the cancer cell lines(A549, AGS, T24, n = 2000/well) were seeded in Opti-MEM® (2.5% FCS, P/S) and after 24 h treated with the respective extract or fractions (c_test_ = 50, 25, and 5 µg/mL, stock solution c = 10 mg/mL in DMSO, max DMSO 0.65%). After additional 72 h, the cells were fixed with chilled trichloracetic acid (V = 100 µL per well). After washing (water, n = 4) the cells were stained with sulforhodamine B (SRB, 0.4% in acetic acid (1%), t = 30 min). Then, the plates were washed (acetic acid 1%, n = 4) and tried under air. The dried dye was dissolved with a TRIS solution (10 mM in water, V = 100 µL per well) and the absorbance measured at λ = 540 nm with a plate reader (Tecan, Spark, M10, Männedorf, Switzerland). Where applicable, EC_50_ values were calculated from biological triplicates with GraphPad Prism for Windows 64-bit Version 10.2.0 (392) (GraphPad Software, Bosten, MA, United States, www.graphpad.com) employing the relative Hill-Slope equation and is given with its confidence interval (95%).

### Tubulin polymerisation in vitro assay and in silico pharmacophore screening

#### Tubulin polymerisation assay

The assay test kit, Tubulin polymerisation HTS assay using > 99% pure tubulin, fluorescence-based (#BK011P), was purchased from Cytoskeleton, Inc. (Cytoskeleton Inc., Denver, CO, USA) (www.cytoskeleton.com)^21^.

The fluorescence-based tubulin polymerisation experiment was performed as previously described ^22^ and in line with the kit manual,applying some minor modifications. Each kit component was reconstituted as a solution and stored as described in the kit guide ^21^. To prepare the tubulin stock, lyophilised brain tubulin powder (> 99%, porcine, m = 10 mg) was placed on ice. The tubulin powder was resuspended in the supplied supplemented buffer 1 (V = 1.1 mL) and kept on ice (t = 2 min) to ensure complete resuspension. On ice, the tubulin stock (c = 10 mg/mL, V = 88 μL) was dispensed as aliquots into labelled Eppendorf tubes (0.5 mL) and snap-frozen with liquid nitrogen. The tubulin stock was stored at T =   -80 °C until later use. To prepare the tubulin reaction mix for each assay, the GTP stock solution (c = 100 mM, V = 20 μL) and buffer 1 (V = 1.5 mL) were thawed and placed on ice. Glycerol buffer was placed on ice. Next, the tubulin stock was thawed and immediately placed on ice, and the mix components were combined as follows: buffer 1 (V = 205 µL), glycerol buffer (V = 150 μL), GTP stock solution (V = 4.4 μL), and tubulin stock (c = 10 mg/mL, V = 85 μL) and kept on ice. Test compounds and control solutions were prepared by dissolving samples in DMSO (c = 3 mM), and from this, aqueous stock solutions (c = 300 μM) were prepared by adding the DMSO/compound stock (V = 100 μL) to Milli-Q water (V = 900 μL).

For screening, each stock solution (V = 5 µL) was added to separate wells of the assay plate provided with the kit (Cytoskeleton Inc., Denver, CO, USA; Half area 96-well plate, black, flat bottom, Corning®, Glendale, AZ, USA) with final DMSO concentrations (< 2%). The 96-well plate was submitted to a Tecan Spark 10 M plate reader (Tecan, Männedorf, Switzerland) to warm it (t = 1 min, T = 37 °C). Then, the tubulin solution (V = 50 µL) was pipetted into each well. The final test compound concentration in the wells was c = 30 μM. The fluorimeter function equipped with filters was preset to excitation at λ = 340 nm (20 nm bandwidth), and the monochromator was set to emission at λ = 450 nm. The gain was manually set to 40 (mirror, flashes = 30, integration time = 40 μs, lag time = 0 μs). The reaction plate was resubmitted to the temperature-controlled plate reader (t = 60 min, T = 37 °C). The polymerisation inhibitors colchicine (TCI Chemicals, Eschborn, Germany, c = 3 µM) and the tubulin stabiliser paclitaxel (Cytoskeleton, Inc, Denver, USA, c = 3 µM) were used as the positive controls.

#### Pharmacophore-based fitting

Pharmacophore models for the taxane binding site (unpublished data) were created in an established pharmacophore modelling workflow analogous to Horgan et al. ^22^. Structure-based models were created in LigandScout (LS) 4.4.5 (www.inteligand.com) ^23^ and optimized to find a dataset of selective taxane binding site tubulin polymerisation enhancers from the literature (n = 56). The model used in this study was based on PDB entry 5MF4 ^24^.

## Results and discussion

To unravel the anthelminthic activity of *C. alpina,* several biological activity investigations (embryo development, worm motility, cytotoxicity) as well as phytochemical analysis of the liquid–liquid derived subfractions were performed. Thereafter, the most bioactive fractions identified were separated further by FCC and SEC fractionation to yield enriched subfractions and pure compounds that were resubmitted to the anthelmintic bioassays for screening alongside compound chemical characterisation. Lastly, to substantiate if the cause of embryo inhibition by *C. alpina* phytochemicals, was alike to the tubulin destabilising mechanism of the benzimidazole controls, samples were submitted to a tubulin polymerisation assay to assess activity directly against the target.

### Embryo development assay with *A. galli*

**Fig. 1:**
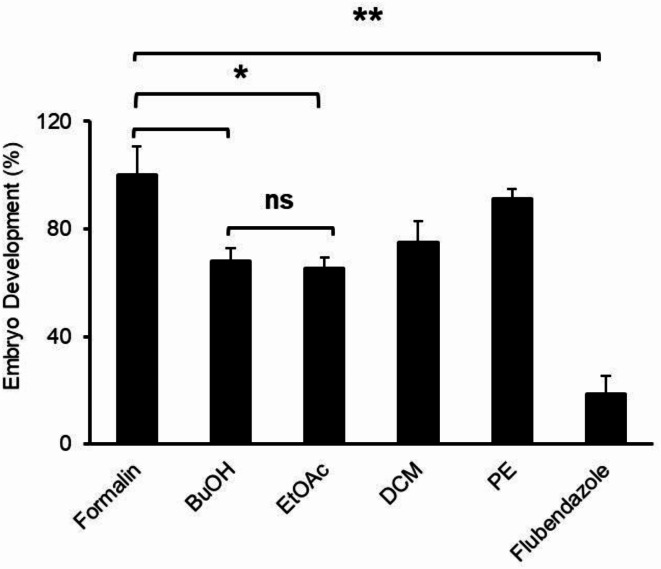
Percentage of developed *A. galli* embryos expressed as % of formalin control (Mean ± SEM) during 28 days exposure to the *C*. *alpina* liquid–liquid fractions, positive control (Flubendazole, c = 1.6 µM), and negative control (Formalin, 0.5%). Mean values (n = 2) were compared by unpaired t-test (* = *P* ≤ 0.05, ** = *P* ≤ 0.01, *** = *P* ≤ 0.001, n.s = not significant).

The *C. alpina* MeOH extract was subjected to liquid–liquid fractionation because of its promising anthelmintic activity previously reported ^15^. In the embryo development assay, the liquid–liquid fractions (*BuOH*, **EtOAc**, **DCM**, **PE**) derived were screened to determine their *A. galli* inhibitory activity at c = 500 µg/mL test concentrations (Fig. 1).

The positive control flubendazole showed a significantly lower level of embryonic development compared to all tested samples. Flubendazole as an anthelmintic drug disrupts embryo cell division through inhibiting microtubule formation by interacting at the tubulin colchicine binding site ^25^. The apolar DCM and PE fractions displayed weak to no activity with 75.0% and 91.2% embryo development respectively. Meanwhile the EtOAc and polar BuOH fractions showed promising activity with 65.5% and 68.0% embryo development respectively, and within activity range of the previously reported MeOH extract at the same concentration ^15^.

Despite its demonstrated robustness, the major drawback of the embryo development assay is its limited throughput, due to the long embryogenesis of *A. galli* ^20^; 28 days are needed for one run. Moreover, the ascarid egg sample accessibility (host infections) ^26^ is fluctuating. Furthermore, in-depth morphological embryo development assessment requires laborious efforts for the assay operator to determine the effect on the phenotype under the microscope. To circumvent this, an additional ascarid worm motility (WMT) phenotype model was considered. However, ascarid worms are known for complex culturing and maintenance under in vitro conditions ^10,11^. Thus, the model organism *C. elegans* and adult WMT phenotype with the automated WMicrotracker was introduced to the workflow as a complimentary screening tool to further prioritise and guide fractionation.

### Worm motility assay (WMT) in WMicrotracker with *C. elegans*

**Fig. 2:**
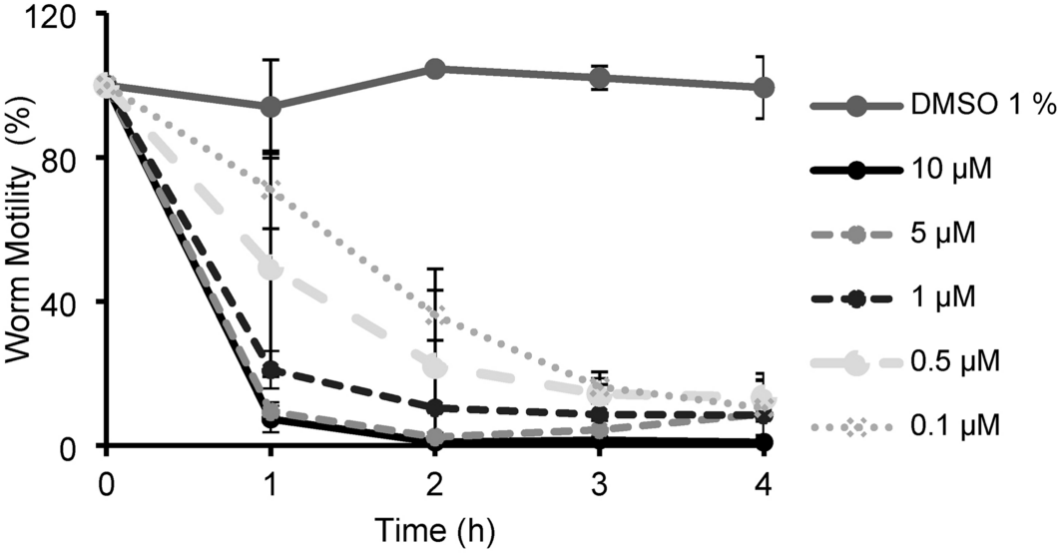
The concentration (c = 0.1—10 µM) and time dependant effect of ivermectin on motility of *C. elegans* adult worms measured with the WMicrotracker. Data normalised to the baseline and values expressed as a % of DMSO vehicle control (Mean ± SD). Measurements performed in duplicate as biological replicates (n = 2).

To first establish and validate the worm motility assay, *C. elegans* adult worms were treated with the vehicle control (1% v/v DMSO) and various concentrations of the anthelmintic ivermectin (c = 0.1, 0.5, 1, 5, and 10 µM) (Fig. 2). From another study published ^27^, it is known that the adult thrashing rate (body bends per second) in liquid significantly decreased (by 29%) with 1.5% DMSO administration and only slightly decreased (less than 2%) with 0.5% DMSO. In our setting, worms exposed only to the vehicle control (1% DMSO) maintained motility and had visibly bending and motile bodies when viewed under the microscope after the experiment (SI, Sect. 2.1.1, Figure S1). Therefore, the effect on motility with sample vehicle control at 1% DMSO was determined to be viable for further experiments. For ivermectin, the lowest concentration (c = 0.1 µM) showed reduced adult motility with 71% within one hour and then just 36% by two hours (Fig. 2). A similar effect was previously shown, as a 50% reduced motility (EC_50_ = 0.2 µM) was reported after 1.5 h ^28^. By four hours, all ivermectin concentrations showed dramatically reduced average motility with values < 13% WMT (Fig. 2). After exposure, worm bodies were visibly rigid and paralyzed under the microscope (SI, Sect. 2.1.1, Figure, S1). The results demonstrated a clear concentration and time dependant effect on adult worm motility up to four hours. Thus, a 1-to-4-h time interval was chosen as a suitable time point range for screening *C. alpina* fractions for effect on the worm motility phenotype. Overall, this confirmed the validity of the motility device and suitability of ivermectin as a positive control as with previous insights ^28^. The preliminary worm motility results (SI, Sect. 2.1.1, Figure S2) revealed the parent MeOH extract (c = 150 µg/ml) to have a mild effect compared to the vehicle control between 1.5–3 h, with its highest motility reduction observed at two hours with 67.4% worm motility.

**Fig. 3:**
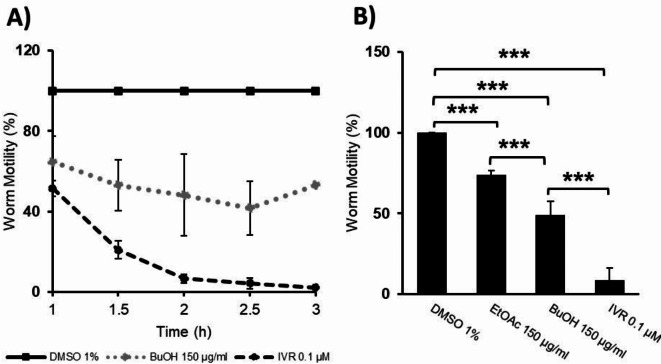
**A**) The effect of fraction BuOH (150 µg/ml), and ivermectin (IVR, 0.1 µM) on worm motility over 3 h measured with the WMicrotracker. **B**) The effect of fractions BuOH (150 µg/ml) and EtOAc (150 µg/ml) on relative worm motility averaged between time points 1.5–3 h. Motility expressed (Mean ± SEM) as % of DMSO control. Measurements performed as biological triplicates (n = 3) with internal technical replicates for the fractions (n = 3, n = 4, n = 3) and controls (n = 6, n = 6, n = 6). Mean values were compared by one-way ANOVA with the Tukey post hoc test (* = *P* ≤ 0.05, ** = *P* ≤ 0.01, *** = *P* ≤ 0.001).

Next, soluble fractions (EtOAc, DCM, BuOH) of the MeOH extract were tested at c = 150 µg/ml. The apolar PE fraction was omitted due to solubility limitations under the test conditions (1% DMSO). From the results (SI, Sect. 2.1.1, Figure S2), the DCM fraction was considered inactive with 92–100% worm motility. The EtOAc fraction had a mild effect (36% reduction) on motility over 1.5–3 h. The BuOH fraction had the strongest effect on *C. elegans* adult worm motility with an average of 49% worm motility between 1.5–3 h (Fig. 3). Moreover, the BuOH fraction showed a slight time-dependant effect with a gradual decrease in motility observed from 1 h (WMT = 64.8%) to 2.5 h (WMT = 41.7%). As the most active sample in the worm motility assay, the BuOH fraction was subsequently tested at different concentrations (c = 50, 100, and 150 µg/ml) and showed worm motility inhibition in a concentration dependent manner (SI, Sect. 2.1.1, Figure S3).

These promising worm motility inhibition results revealed that the BuOH fraction of *C. alpina* was bioactive against the *C. elegans* adult worms and suggest another plausible helminth phenotype to target in addition to the *A. galli* embryo results. Thus, it could be considered as a dual anthelmintic. Similarly, a liquid–liquid derived *Typha capensis* BuOH fraction showed dual anthelmintic activity with 50% egg hatch inhibition (EC_50_ = 500 µg/mL) against *H. contortus* eggs and larval development (EC_50_ = 311 µg/mL) after 24 h, and against *C. elegans* it was more active than the positive control levamisole after 72 h ^29^.

### Cytotoxicity assay with human cell lines

With the dual anthelmintic activities of the CA_MeOH extract and BuOH fraction identified, the potential adverse effect of these substances to a non-helminth chordate host organ cell was considered. As human cancer cell lines are rapidly dividing, they were chosen as a counter screening platform to the rapidly dividing embryo cells within the ascarid eggs to differentiate selective or ubiquitous toxicity between helminth and non-helminth eukaryotic cells.

Thus, the MeOH extract liquidl-iquid fractions (PE, DCM, EtOAc, and BuOH) were submitted to a cytotoxicity assay with albenazole (TCI Chemicals, Zwijndrecht, Belgium) as the positive control, and results are summarised below in Table 1. Unsurprisingly, the albendazole control was highly potent against all cell lines with low EC_50_ values (i.e., EC_50_|A549| = 1.7 µM, EC_50_|AGS| = 0.7 µM, and EC_50_|T24| = 0.4 µM). As an anthelmintic and antiproliferative agent, albendazole’s mechanism of action involves disrupting mitosis by inhibiting microtubule formation through interactions at the tubulin colchicine binding site alike to flubendazole^25^. The MeOH extract had low to no cytotoxicity with high EC_50_ values (i.e., EC_50_|A549| > 50 µg/mL, EC_50_|AGS| > 50 µg/mL, and EC_50_|T24| = 50 µg/mL).

**Table 1:** Cytotoxicity activities and EC_50_ values of CA_MeOH extract and *C.* *alpina* liquid–liquid fractions against cells of human lung (A549), stomach (AGS), and bladder (T24) cancer cell lines compared to positive control albendazole.

			Cell line			
EC_50_ (CI 95%)	A549 (lung)		AGS (stomach)		T24 (bladder)	
Control Albendazole (µM)	1.7	3	0.7	0.4	0.4	0.3
		1.1		0.2		0.2
**Extract**						
MeOH (µg/mL)	> 50		> 50		50	7.5
						7.5
**L-L fraction**						
PE (µg/mL)	> 50		> 50		> 50	
DCM (µg/mL)	~ 25		9.6	1.2	11.8	0
				1.2		0
EtOAc (µg/mL)	~ 25		11	2.2	18.7	2.7
				2.2		2.7
BuOH (µg/mL)	> 50		> 50		> 50	

Among the *C. alpina* fractions, the DCM sample exhibited the strongest toxicity and was the most potent with low EC_50_ values across all cell lines (i.e., EC_50_|A549| = 25.2 µg/mL, EC_50_|AGS| = 9.6 µg/mL, and EC_50_|T24| = 11.8 µg/mL). The second most toxic fraction was EtOAc (i.e., EC_50_|A549| = 25.9 µg/mL, EC_50_|AGS| = 11 µg/mL, and EC_50_|T24| = 18.7 µg/mL). The more apolar fraction (PE) demonstrated low cytotoxicity against all three-cancer cell lines (i.e., EC_50_|A549| > 50 µg/mL, EC_50_|AGS| > 50 µg/mL, and EC_50_|T24| ~ 75 µg/mL). The BuOH sample showed low cytotoxicity (i.e., EC_50_|A549| > 50 µg/mL, EC_50_|AGS|  > 50 µg/mL, and EC_50_|T24| ~ 60 µg/mL), thus was the most promising result among the liquid–liquid fractions, as the probability of adverse effects is lowest. Interestingly, one study also found that a BuOH fraction containing flavonoid glycosides was non-cytotoxic to monkey kidney cells while displaying anthelmintic activity ^29^.

### Phytochemical analysis of the bioactive liquid–liquid fractions

Preliminary HPLC-DAD and HPLC-DAD-MS analysis of the bioactive DCM, EtOAc, and BuOH fractions revealed both identical and ubiquitous major peaks eluting from the fractions detected at the 254 nm wavelength (SI Sect. 2.2.1, Figure S13)**.** A summary of the retention times, UV-Vis absorbance, values, and MS molecular ion peaks are shown in the supporting information, Tables S1 and S2.

For BuOH, an early eluting peak at retention time of t_R, Peak 1_ = 9.8 min was annotated as chlorogenic acid based on similar retention time previously reported under similar experimental conditions ^30^ and its UV–Vis typical absorbance band ^31^. Furthermore, the retention time correlated with that of the standard reference compound and with a positive mode mass/charge ratio of [M + H]^+^ = 355.15 m/z it could be assigned to this mono-*O*-caffeoylquinic acid with a mass of M = 354.1 g/mol. Interestingly, the compound was previously reported as anthelmintic with an in vitro ovicidal effect (LC_50_ = 248 μg/mL) in an egg hatch inhibition assay ^32^. Such findings hint towards the speculation that this caffeic acid derivative could be partly responsible for the anthelmintic activity observed for the BuOH fraction in this study.

Next, a major peak at t_R, Peak 2_ = 12.2 min present in the DCM, EtOAc, and BuOH fractions with an accompanying UV–Vis absorbance (SI Sect. 2.2.1, Table S1) revealing one broad band at λ_max_ ~ 258 nm (250–270 nm). This characteristic spectrum suggested the presence of a sesquiterpene lactone due to the chromophore (the alpha–beta unsaturated carbonyl group) typically absorbing light in this wavelength range. Furthermore, the identified peaks in both fractions had a positive mode mass/charge ratio of [M + H]^+^  = 279.10 m/z. Comparing to the literature, the compound 11β,13-dihydrolactucin contains this precursor ion ^33^**.** ^1^H and ^13^C NMR data confirmed the structure. Specifically, stereochemistry at position 11 can be assigned by coupling constant ^1^H-^1^H between H-11 and H-7. In case of *trans* configuration, a coupling constant of *J* = 9–12 Hz should be observed, and in case of *cis* configuration a coupling constant of *J* = 6–8 Hz should arise. 11β stereochemistry can be unequivocally assigned by coupling constant *J* = 11.9 Hz observed in the ^1^H spectra (SI, Sect. 2.2.2). The compound was previously isolated from *C. intybus* but reported inactive against *A. suum* larvae ^34^.

Another major peak in the comparatively apolar DCM fraction detected at retention time of t_R, Peak 9_ = 34.6 min was not found in the more polar EtOAc and BuOH samples. The peak’s UV–Vis trace had one broad band at λ_max_ ~ 257 nm and MS analysis showed a positive mode mass/charge of [M + H]^+^  = 261.14 m/z, thus tentatively assigning it to the sesquiterpene lactone 8-deoxylactucin, as previously annotated in *C. alpina* and related *C. intybus* extracts ^35,36^. A positive correlation between increasing concentrations of 8-deoxylactucin in *C. intybus* extracts with *H. contortus* egg hatch inhibition has been shown, with 1.3 mg/ml of the compound (extract LC_50_ = 2.6 mg/ml) contributing to activity ^36^. While another study confirmed *A. suum* larvae mortality (EC_50_ = 85 μg/mL) ^34^. Therefore, it could be speculated that the 8-deoxylactucin content within the DCM fraction was not sufficiently enriched to induce an anthelmintic effect in the WMT or embryo development assays at the test concentrations (c = 150 µg/ml and 500 µg/ml). However, the presence of this compound likely contributed to the DCM fraction’s high cytotoxicity, as it has been previously reported as cytotoxic to HeLa cells (ED_50_ = 0.26 μg/ml) at low concentrations ^37^. A tentative assignment was also made for the DCM fraction peak t_R, Peak 12_ = 44 min, as the furanocoumarin ostruthol (M = 386.14 g/mol, [M + H]^+^  = 387.3 m/z), detected previously in *C. alpina* root extracts ^38^. The presence of substances from this compound class could co-explain the observed mammalian cell toxicity as their cytotoxic activities are well documented ^39^.

### Bioactivity evaluation and phytochemical analysis of BuOH and EtOAc subfractions

To further gain insight into the active phytochemical principle of the most interesting fractions (i.e., the non-cytotoxic and anthelminthic BuOH fraction as well as the anthelmintic EtOAc fraction) eight subfractions of the BuOH fraction (**B1**–**B8**) and thirteen EtOAc subfractions (**E1**–E**13**) were obtained via silica-gel flash column chromatography (FCC). Additional eight subfractions derived from **E3** (**E3.1**–**E3.8**) were obtained with size exclusion chromatography (SEC). All obtained subfractions were submitted to HPLC–DAD measurements (SI, Sect. 2.2.1, Figures S13, S14, and S15) and to the respective bioassays were possible and meaningful.

### Worm motility assay (WMT) in WMicrotracker with *C. elegans*

The WMT assay results (SI, Sect. 2.2.1, Figure S4) showed that the samples (**B1** to **B7,** **E2** to **E4**, and **E7** to **E13**) were all considered to be inactive (WMT  > 79–100%). The samples (EtOAc, **E1**, and **E5/E6**) were considered as mildly active (WMT = 61–78%) and the samples (**BuOH** and **B8**) were regarded as highly active (WMT = 0–60%). The most prominent anthelmintic effect was observed for the **B8** fraction derived from the BuOH fraction with the bioactivity range retained at the same concentration (**B8** |58% WMT| c = 150 µg/ml). Thus, it could be considered that no loss in activity occurred during fractionation as so often encountered. Furthermore, a preliminary study (one biological replicate, 3 technical replicates) comparing the active BuOH and **B8** fractions was performed at c = 500 µg/ml (SI, Sect. 2.1.1, Figure S5). Results at 500 µg/ml showed the arbitrary activity was within range of ivermectin/levamisole controls. Phytochemical analysis revealed the following insights for the **B8** fraction: (i) Peak 1, at retention time of t_R, Peak 1 =_ 9.8 min, was annotated as chlorogenic acid, (ii) Peak 8 eluting at t_R, Peak 8_ = 31.6 min was assigned to 3,5-dicaffeyolquinic based on mass, UV–Vis spectra, and the retention time, (iii) peak 10, at a retention time of t_R, Peak 10_ =  ~ 34.8 min, was tentatively assigned to 4,5-dicaffeoylquinic acid with a mass of M = 516.12 g/mol. Derivatives of this phenolic family have been previously declared in *C. alpina* extracts ^15,30,40^. Moreover, with the caffeoylquinic acid rich **B8** subfraction identified active in the worm motility study, this result corresponds with previous findings already confirming the anthelmintic effect of this phytochemical class on *H. contortus* larvae and eggs ^32,41,42^. Contrastingly, caffeoylquinic acid derivatives extend *C. elegans* lifespan at 25 µM -100 µM when treated over days ^43,44^. Thus, if an anthelmintic mortality endpoint is not achieved in the assay, caffeoylquinic acid derivatives might promote survival and longevity of parasitic worms.

### Embryo development assay (ED) with *A. galli*

The flash column chromatography and size exclusion chromatography derived subfractions were submitted in parallel for screening in the *A. galli* embryo development assay. Fraction **B2** was omitted from testing due to the low yield and later identical **B1** assignment (SI, Sect. 2.2.1, Figure S13 and Table S1). For the ED screening of the BuOH and EtOAc subfractions, samples were again assayed at c = 500 µg/ml and compared to flubendazole (0.5 mg/ml, 1.6 μM), formalin, and water negative controls. The ED assay results are found in the supporting information (SI, Sect. 2.1.2, Figure S6-S10). The positive control (flubendazole) with 20.7–25.5% ED, showed significantly lower embryonic development compared to all test samples. The ED assay results revealed some test samples as mildly active (**B5**, **B7**, **B8**, **E1**, **E2/E3**, **E5/6**, **E9**, **E12**, **E13**, and **E3.3** | ED = 61–80%) or considered as inactive (**B6**, **E1**, **E4**, **E7**, **E8**, **E3.1**, **E3.2** and **E3.4** | ED =  > 80–100%) when compared to the formalin control.

**Fig. 4:**
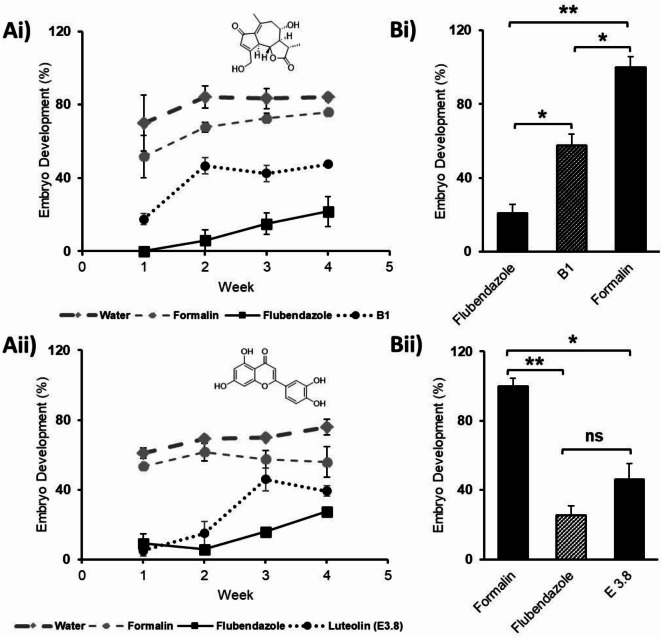
**Ai**) The effect of fractions **B1** and **Aii**) **E3.8 **(luteolin) on *A. galli* embryo development (Mean ± SEM) over 4 weeks compared to the positive control (flubendazole) and negative controls (formalin, water). **Bi)** The effect of **B1** and **E3.8** **Bii**) shown as the mean percentage of developed *A. galli* embryos normalised to the negative control (Mean ± SEM) during 28 days exposure to the test samples compared to the positive control (Flubendazole) and negative control (formalin). Mean values were compared by one-way ANOVA with the Tukey post hoc test (* = *P* ≤ 0.05, ** = *P* ≤ 0.01, *** = *P* ≤ 0.001, ns = not significant).

The subfraction **B3/B4** showed high activity against the developing embryos with 54.7% embryo development. The enriched fraction **B1** (500 μg/mL, 1.8 µM) and luteolin (Thermo Fisher Scientific, Langenselbold, Germany) (500 μg/mL, 1.7 µM) were also highly active with 57.6 and 46.0% ED, respectively. Evaluation of the inhibitory effect of the enriched **B1** subfraction and luteolin (**E3.8** subfraction) on developing embryos over 4 weeks is shown (Fig. 4). The result shows that the samples were comparable to the positive control flubendazole (0.5 mg/ml, 1.6 μM) at different experimental time points. With lower ED compared to the other test samples (SI, Figure S6), **B1 **and luteolin (**E3.8**) had significantly lower percentual embryo development compared to the negative controls (formalin, water, Fig. 4 and SI, Figure S6).

### Cytotoxicity of 11β,13-dihydrolactucin

The **B1** fraction enriched with 11β,13-dihydrolactucin (up to 25 µM) was assessed in the cytotoxicity assay to identify potential acute cytotoxic activity. Against all three tested cell lines (i.e., A549, SV80, and T24) no cytotoxic activity (EC_50_ > 25 µM) was observed. This is in line with previous reports against nasopharyngeal cells (KB) and liver cancer (Bel 7402) cells where the absence of cytotoxic activity was determined ^45^. Therefore, the confirmed non-cytotoxicity of this substance strengthens the use of 11β,13-dihydrolactucin enriched herbal extracts as deworming nutraceuticals.

### Phytochemical analysis of the enriched bioactive fractions

For the **B3/B4** fractions, the major peak present at t_R, Peak 4_ = 31.7 min was tentatively assigned to 8-acetyl-15β-D-glucopyranosyllactucin and its adjacent peak 5 tentatively assigned as an 8-acetyl-glucopyranosyllactucin derivative based on similar retention time and UV–Vis absorbance values (SI, Sect. 2.2.1, Figure S13 and Table S1) to the standard, in addition to literature retention times, peak elution order, and UV–Vis absorbance value ^30,38,46^. One minor peak was detected t_R, Peak 3_ = 34.4 min that could not be assigned, however, its UV–Vis spectra revealed one sharp band at 240 nm and one broad band at 270 nm, suggesting the presence of a sesquiterpene lactone. Furthermore, the peak’s later elution time is possibly owed to one of the less polar lactucin derivatives that have been reported to elute later such as; 13-dihydro-8-deoxylactucin, 11,13-dihydro-lactucopicrin, and lactucopicrin ^33,40,47–49^.

For the **B1** fraction, the single purified Peak 2 = 12.2 min was assigned to 11β,13-dihydrolactucin when compared additionally to the isolated internal standard with comparable NMR, Mass, UV–Vis (SI, Sect. 2.2.1 and 2.2.2), and literature values ^33^.

In **E3.8**, the late eluting substance with the retention time t_R, Peak 11_ =  ~ 38 min and a positive mode mass/charge ratio ([M + H]^+^  = 287.1 m/z) was detected (SI, Sect. 2.2.1, Figure S15 and Table S1/S2). Moreover, the UV-Vis trace of Peak 11 is characterised by two main peaks, one double-peaked band at λ = 254 nm and a broad peak at λ  = 349 nm. This pattern and mass suggested the presence of a hydroxylated flavonoid and confirmed as luteolin by NMR and co-eluting retention time (SI, Sect. 2.2.1 and 2.2.2) with the luteolin (Thermo Fisher Scientific, Langenselbold, Germany) standard.

The **B1** fraction was inactive against *C. elegans* WMT and thus potentially excludes the adult worm motility phenotype as a viable helminth lifecycle stage to target for 11β,13-dihydrolactucin. Previously, Valente et al. revealed that 11β,13-dihydrolactucin was inactive at 500 μg/mL against *A. suum* larvae after 24 h ^34^. This hints that there is molecular target expressed more abundantly within the developing embryo than in the worm itself. Moreover, the observed toxicity for **B1** against parasite embryo cells and not human cells suggests this compound could be more selective to parasites and not to the host.

### Egg hatch assay with *C. elegans*

**Fig. 5:**
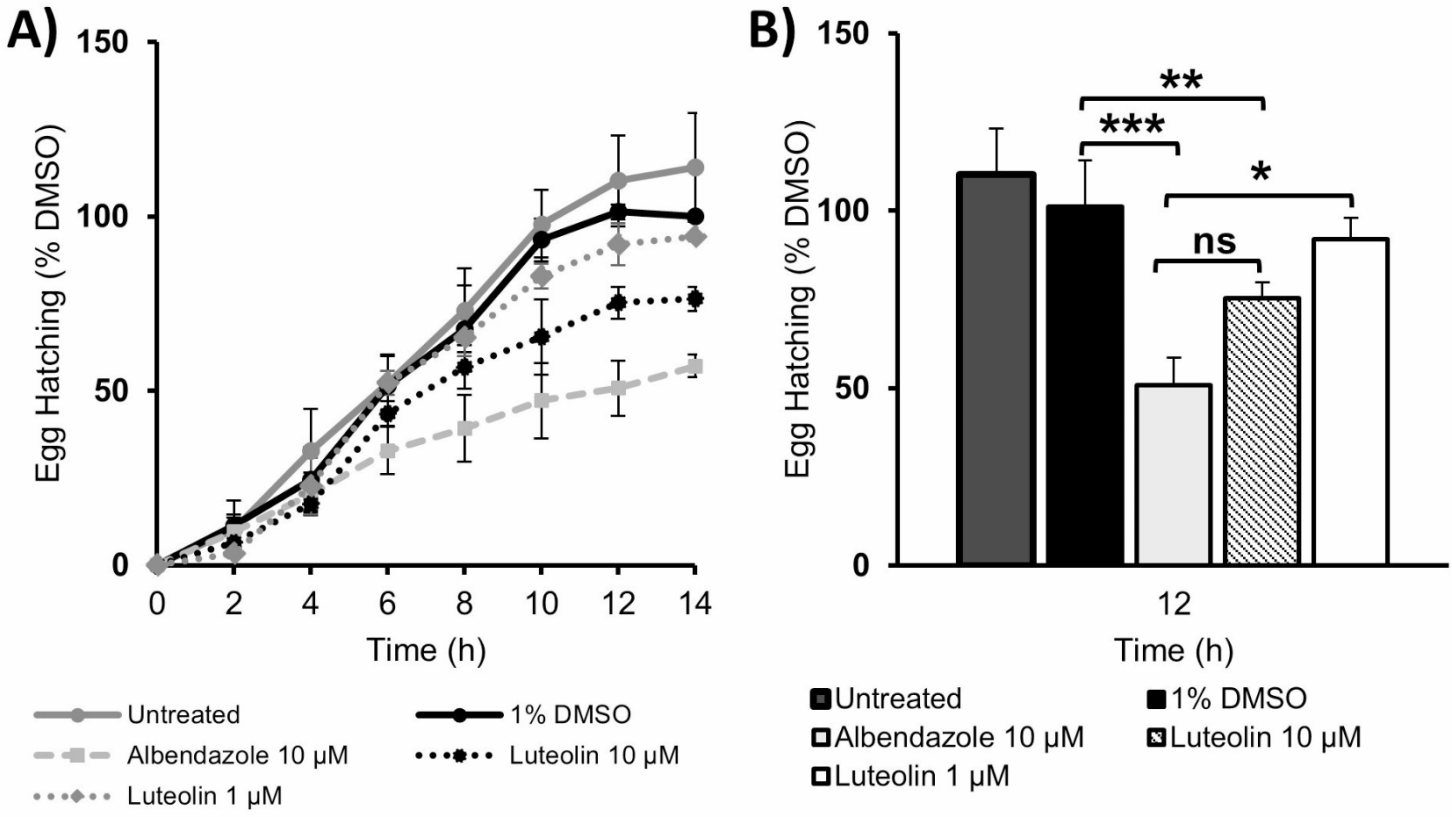
**A)** Percentage egg hatching of *C. elegans* embryos over time. Data normalised to the DMSO (1%) control at time point 14 h with values expressed (Mean ± SEM). Repeat measurements for the controls were performed as biological replicates (n = 4), with internal technical replicates: albendazole (n = 5, n = 10, n = 4, and n = 6), 1% DMSO (n = 5, n = 5, n = 4, n = 6), and untreated (n = 5, n = 5, n = 3, n = 30). Measurements for luteolin (c = 10 µM) were performed in biological duplicate (n = 2) and luteolin (c = 1 µM) in biological triplicate (n = 3), with internal technical replicates (n = 3, n = 6) for both. **B)** The effect of samples on egg hatching at time point 12 h compared to controls. Mean values were compared by unpaired t-test (* = *P* ≤ 0.05, ** = *P* ≤ 0.01, *** = *P* ≤ 0.001, ns = not significant).

To determine whether the observed embryo development inhibition effect of luteolin (**E3.8**) is not only limited to the ascarid *A. galli* eggs but ubiquitously active across other species of helminths, a complimentary assay measuring *C. elegans* egg hatching (% EH) with the WMicrotracker was employed. The anthelmintic albendazole (c = 10 µM) and DMSO (1%) were used as the positive and negative controls, respectively. The egg hatching assay results are presented below in Fig. 5. Albendazole (c = 10 µM) proved to be a suitable positive control and clearly demonstrated egg hatch inhibition over time (t = 10 h | 47.2% EH). When luteolin (Thermo Fisher Scientific, Langenselbold, Germany) (c = 1 µM) was tested at the comparable concentration to the embryo development assay, little effect on *C. elegans* hatching was observed. However, the flavonoid did exert an anthelmintic effect when treated at a tenfold higher concentration (c = 10 µM), also demonstrating its egg hatch inhibition effect around 9–12 h (t = 10 and 12 h | 65.4 and 75.3% EH) compared to the DMSO control. Significance determined by unpaired t-test (* = *P* ≤ 0.05, ** = *P* ≤ 0.01, *** = *P* ≤ 0.001, n.s = not significant).

The *C. elegans* egg hatch inhibition activity of albendazole is a well-documented phenotype for this benzimidazole derivative ^50^, with its ovicidal mechanism of action alike to flubendazole via mitotic cell arrest through tubulin inhibition ^25,50^. As an anthelmintic, luteolin has shown *S. mansoni* trematocidal activity (t = 6 h | IC_50_ = 4.6 μg/mL), (t = 12 h | IC_50_ = 5.8 μg/mL) and, *T. muris*, motility reduction (IC_50_ = 9.7 μg/mL) respectively ^51^. However, its anthelmintic effect on the embryo life cycle stage has not been previously confirmed or elucidated.

Respectively, worms are not the sole phenotypic target of this phytochemical class, as flavonoids have demonstrated in vitro ovicidal properties against a plethora of helminth species. The compound quercetin inhibited both embryo development and hatching of *Fasciola hepatica* eggs ^52^. Moreover, Yamssi and colleagues ^53^ suggested that the observed ovicidal effect of extracts rich in flavonoids might be due to entry through the eggshell, preventing the segmentation of blastomeres or perhaps paralyzing the developed larvae inside embryonated eggs and inhibit hatching ^53^.

In terms of applications, this method and instrument could be adapted to analyse egg hatching of other parasitic helminths as demonstrated in a *Heligmosomoides polygyrus* egg hatch model ^53^. Furthermore, the rapid embryogenesis cycle and hatching of *C. elegans* eggs typically has a much shorter time frame (~ 13–14 h) compared to ascarid egg development (~ 28 days) ^20^. Thus, establishment of this egg hatching assay as phenotypic model assisted with fortifying results from the embryo development assay, offering an advantageous future option in terms of high throughput for selection and prioritisation of anthelmintic extracts and fractions.

With luteolin invoking its inhibitory effect in a comparable manner and concentration range to both benzimidazole positive controls in both egg assays, it could be speculated that this flavonoid elicited its anthelmintic effect by interacting with the same tubulin target within the helminth embryos. To test this hypothesis, the isolated compounds were submitted to an in vitro tubulin polymerisation assay.

### Tubulin polymerisation assay and virtual screening

The tubulin assay was established previously and successfully confirmed the validation of a pharmacophore workflow targeting the colchicine binding site of tubulin, leading to the identification of novel inhibitors ^22^. We anticipated that the colchicine binding site could likely be the main tubulin site of interaction, owed to prior work demonstrating that the analogous anthelmintic flavonoid quercetin inhibits tubulin polymerisation (IC_50_ = 54 µM) and specifically inhibits colchicine binding to tubulin but does not bind at the vinblastine site ^54^. Moreover, in silico molecular docking and dynamics simulations at the tubulin colchicine binding site suggest that flavonoids could be excellent anti-tubulin agents ^55^. In the assay, colchicine (c = 3 µM) and paclitaxel (c = 3 µM) were used as positive controls and an untreated blank as the negative control.

Compared to the untreated blank, unsurprisingly, the fluorescence readout from colchicine had a decreased signal intensity, while paclitaxel showed a sharp and early increase in the signal (SI, Sect. 2.1.3, Figure S11). The test samples luteolin (Thermo Fisher Scientific, Langenselbold, Germany) (c = 50 µM) and 11β,13-dihydrolactucin (c = 50 µM) both caused an enhancement in tubulin polymerisation compared to the blank, with > 25% and > 30% increase in polymerisation respectively at time point 27 min (SI, Sect. 2.1.3, Figure S12).

For luteolin, this was a contrasting result to that already reported for quercetin in the same concentration range, resulting in 50% decreased polymerisation by colchicine binding site interactions ^54^. However, one study found that the flavonoid fisetin stabilises microtubule assembly in vitro and enhanced tubulin polymerisation to a greater extent than paclitaxel ^56^. Thus, from this readout it was predicted that the compounds may interact with tubulin via interacting at the taxane binding site, rather than the colchicine binding site.

**Fig. 6:**
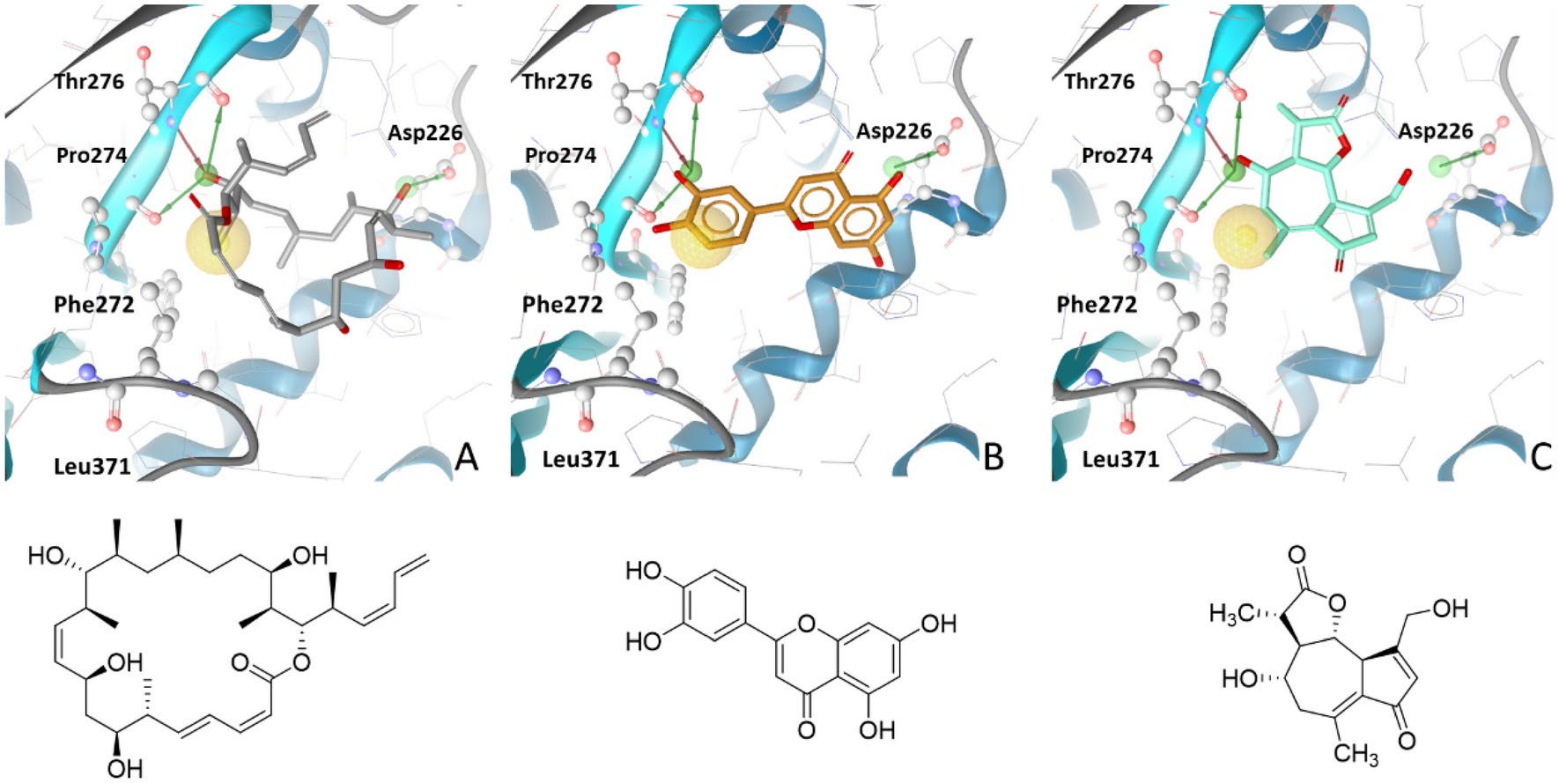
Structure-based pharmacophore model created from the tubulin dictyostatin complex (A ^24^). Key hydrogen bond donor interactions to Asp226, Thr276, and Pro274 are marked with green arrows, hydrogen bond acceptor features to Thr276 are represented with a red arrow, and hydrophobic contacts to Phe272 and Leu371 with a yellow sphere. Luteolin (B) and 11β,13-dihydrolactucin (C) both match the pharmacophore pattern and can therefore form similar interactions in the taxane binding site.

A structure-based pharmacophore model based on dictyostatin co-crystallised with β-tubulin (PDB entry: 5MF4) ^24^ found the two experimentally investigated tubulin enhancers luteolin and 11β,13-dihydrolactucin, illustrating their 3D structural similarities with known tubulin polymerisation enhancers. The model (Fig. 6) consisted of three hydrogen-bond donor features marking interactions with Asp226, Thr276 and Pro274. An additional hydrogen-bond acceptor feature with Thr276 and a hydrophobic feature complete the model. The pharmacophore alignment of luteolin (Fig. 6B) and 11β,13-dihydrolactucin (Fig. 6C) shows that these structures offer hydroxy groups as interaction partners in a similar distance as in dictyostatin (Fig. 6A), even though they represent completely different active scaffolds. The proposed binding mechanisms would explain the experimentally observed activity.

In contrast, correlations between helminth egg hatch inhibition and mammalian tubulin polymerisation inhibition by benzimidazole derivatives has been investigated ^57^. Owed to the fact that helminth embryogenesis in the egg relies on microtubules during mitotic spindle fibre formation ^58^, it can be postulated that antimitotic cell division disruption through tubulin stabilisationis one possible mechanism of action responsible for the observed embryo development and egg hatching inhibition activity of luteolin (**E3.8**) and dihydrolactucin (**B1**) in this study.

## Conclusion and outlook

In this study, natural products derived from a *C. alpina* methanol extract demonstrated in vitro anthelmintic activity against two helminth species, *A. galli* and *C. elegans.* The compounds luteolin and dihydrolactucin were isolated and demonstrated as two of the major active principles responsible for the observed bioactivity. Furthermore, tubulin formation inhibition via polymerisation enhancement of these compounds was shown in vitro with interactions at the taxane binding site predicted by in silico pharmacophore based virtual screening. This result suggests a potential target and possible mode of action for the observed helminth egg hatch and embryo inhibitions.

Considering the outlook, this research further advocates for the pharmacological and nutraceutical use of *C. alpina* extracts and phytochemicals. Cultivation programmes will be considered to breed a variety optimised for prophylactic or deworming applications in livestock or humans. The wider scope will contribute toward the selection and prioritisation of an extract favourable for such purposes by targeted plant cultivation and genetic breeding for an extract with specifically enriched and excluded metabolites.

## Supplementary Information

Supplementary Information.

## Data Availability

Most of the data generated or analysed during this study are included in this published article and its supplementary information files. Missing parts are available from the corresponding author on reasonable request.
