# Supplementary material for: Microtubule inhibition as a proposed mechanism for the anthelmintic effect of phytochemicals isolated from Cicerbita alpina
Source: Sci Rep. 2025 Feb 3;15:4108. doi: 10.1038/s41598-024-73958-9 (PMC11791071; doi:10.1038/s41598-024-73958-9)
Supplement: Supplementary file 1 — Supplementary Information. [file 41598_2024_73958_MOESM1_ESM.docx]

**Supporting Information (Supplementary)**

1. **Methods - Anthelmintic Bioassays**

**1.1 Preparation and culturing of *C. elegans* worms for WMT assay**

- - 1. OP50^-1^ bacteria culture

An LB agar plate with bacteria colonies of OP50^-1^ *E. coli* freshly streaked from frozen stock was prepared for starter culture. Using a disposable plastic pipette tip, a single OP50^-1^ colony was collected and placed into LB medium (V = 3 mL) with streptomycin (c = 50 μg/mL) in an Erlenmeyer flask (size = 20 mL). The culture was incubated overnight growing at T = 37°C while shaking (250 rpm). The next day, a larger OP50^-1^ culture was prepared (1/1,000 dilution) by transferring the starter culture (V = 300 µL) to LB (V = 300 ml) with streptomycin to a 500 ml Erlenmeyer flask. The bacteria culture was incubated at T = 37°C shaking continuously (250 rpm) for 24 hours. Under aseptic conditions, the LB bacteria culture was transferred to sterile and pre-weighed plastic 250 ml centrifuge bottles and centrifuged at (3,000 × g) for 10 min at 21°C–22°C. The resulting supernatant was discarded and the remaining bacteria pellet spun down/washed twice with sterile H_2_O/S-medium. The final supernatant was aspirated and the bacteria pellet mass (m = 0.62 g) determined by subtracting the mass of the bottle from the mass of the bottle with bacteria. The bacteria pellet (m = 0.62 g) was resuspended by vortexing in S-medium (V = 6.2 ml) as OP50^-1^ food stock to a final concentration of (c = 200 mg/ml) and frozen at T = –20°C until required. The above procedure was repeated when fresh OP50^-1^ pellets were required for feeding worms in subsequent assays.

- - 1. *C. elegans* culturing and worm synchronization

NGM media was prepared under sterile conditions according to established formulations: composition per litre: 3 g NaCl, 2.5 g peptone, 17 g agar, 1 mL of 5 mg/mL cholesterol, 1 mL 1 M CaCl2, 1 mL 1 M MgSO4, 25 mL 1 M Potassium Phosphate Buffer (pH 6.0) and seeded with *E. coli* (strain OP 50-1))

Synchronized worm populations were obtained by an alkaline bleaching method with some modifications^1^. Mixed worm populations (adults/larvae/eggs) were collected from NGM culture plates by washing with sterile H_2_O (V = 8ml) into a falcon tube. The worms/eggs were left to settle at the bottom of the tube, after 20 minutes, 7.5 ml of the supernatant was removed and fresh alkaline sodium hypochlorite solution (3.5 ml sterile H_2_O, 1 mL of bleach (4%) and 0.5 mL of 5 M NaOH, V = 3ml) was added. The suspension was gently vortex for 2 minutes until the absence of dissolved worms was clear under the light microscope. The suspension was spun down for 2 mins (rpm = 1,200) and bleach was removed. The remaining eggs were washed and centrifuged X2 (rpm = 1,200) for 2 minutes with sterile H_2_O (V = 5ml) and a final centrifuge (rpm = 2,000) and wash with S-medium (V = 5 mL) was performed. Finally, eggs were re-suspended in S-medium (V = 20ml) and kept on a rotator (10 rpm) at 20 °C overnight to hatch into L1 larvae (L1s). After 12 h shaking, the L1s were redistributed onto fresh NGM plates seeded with *E. coli* OP50^-1^ and incubated at 20 °C developing into young adult worms after 48 h.

1.2 Isolation of *A. galli* eggs for ED assay

Laying hens were slaughtered at the end of the laying period at the local abattoir in accordance with European and Italian legislation (EC 2009) and live adult *A. galli* worms were recovered from the small intestines. The worms were collected in PBS and transferred to the laboratory. The adult female worms were identified and dissected on the same day and the eggs were isolated from the worm uterus according to published methods.^2,3^ Firstly 250 eggs were placed in Petri dishes in 0.5% formalin solution and each one of the fractions B1 – B3, B4 – B7, B8 and the subfractions, E1 – E4, E10, E12, E13, B3_B4, B6, PE_F, DCM_F, Luteolin, E3.1, E3.2, E3.3, E3.4, were tested in duplicate at concentrations 0.500 mg/ml in Petri dishes with 1% dimethylsulfoxide (DMSO).^4^ Untreated Petri dishes without added reagents, negative controls namely Petri dishes with 0.5% formalin and 1% DMSO and positive controls (flubendazole (FL), c = 0.500 mg/ml) were also performed in duplicate. All Petri dishes were incubated at 24.4 °C at 45% humidity for four weeks and starting from the day of egg isolation (d0) until day 28, the ED (*in ovo* larval development) was assessed every day by examining the morphological characteristics of eggs in all Petri dishes.^3,5^ For each measurement 20 eggs (240 eggs/replicate) were randomly selected and their development stage was evaluated. The examined eggs were classified into eight different development stages (infertile, fertile, early development, vermiform, blastula, gastrula, embryonated, and slender). The percentage of the eggs corresponding to each development class was calculated and following they were grouped as either undeveloped or developed.^3^ In total 12,240 eggs were evaluated.

1. **Results**
   1. **Anthelmintic, Cytotoxicity and tubulin assays**
      1. **WMT assay fractions**

**
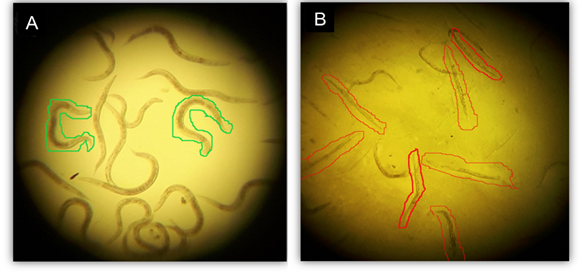
**

Figure S1: Morphological examination after WMT assay: **A)** Normal body curvature of motile worms in 1% DMSO (green) and **B**) rigid body of immotile worms paralyzed (red) after 12 hours exposure to 0.1 µM ivermectin.


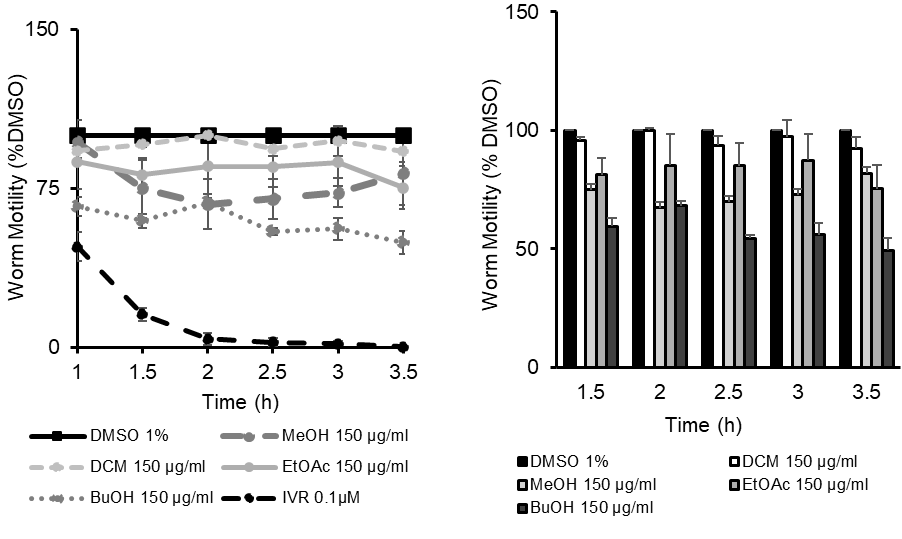


Figure S2: The AH effect on worm motility measured with the WMicrotracker and comparison of Ivermectin, C. alpina MeOH extract, DCM, EtOAc, and BuOH fraction samples (c = 150 µg/ml) over 3.5 hours. Data is expressed as % of the DMSO control (samples n = 4, controls n = 6 with Mean ± SD). IVR = Ivermectin


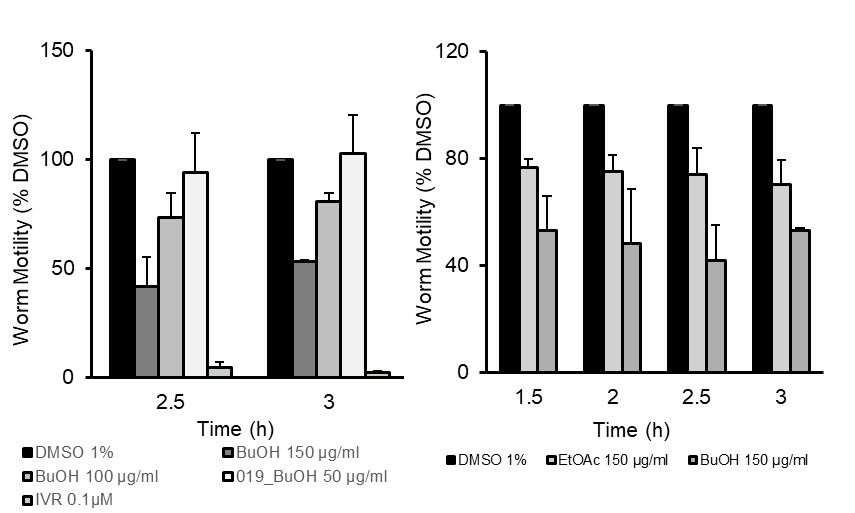


Figure S3: The effect of BuOH-F concentrations (c = 50, 100 and 150 µg/ml) and EtOAc-F (c =150 µg/ml) on worm motility at 2.5 and 3 hours measured with the WMicrotracker. Average motility expressed (Mean ± SEM) as % of DMSO control. Measurements performed as biological triplicates (n = 3) with internal technical replicates for the fractions (n = 3, n = 4, n = 3) and controls (n = 6, n = 6, n = 6).


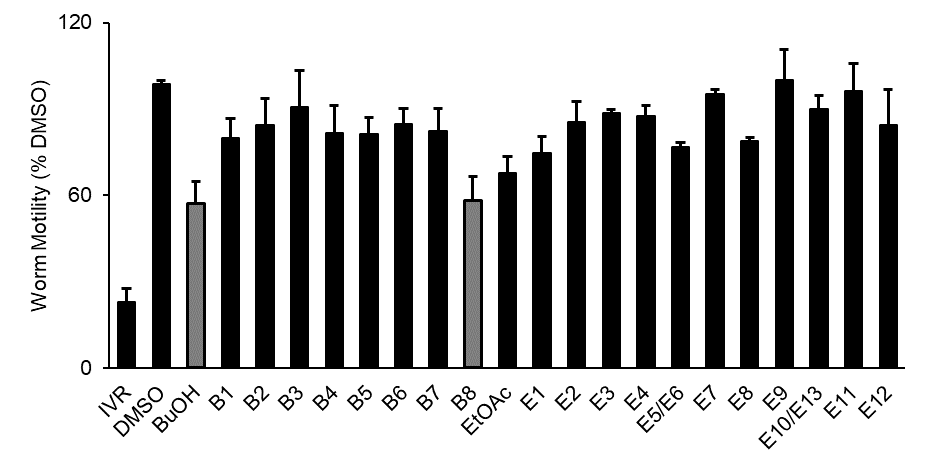


Figure S4: The effect of BuOH-F and EtOAc-F subfractions (c = 150 µg/ml), or where insoluble (c = 75 µg/ml), on worm motility after 4 hours measured with the wMicrotracker. Average motility over time points (Mean ± SEM) expressed as % of DMSO control. Measurements for subfractions were performed as biological duplicates (n = 2) with internal technical replicates for the fractions (n = 3, n = 4, n = 3) and biological replicates for the controls (n = 8). Measurements for fractions performed in duplicate BuOH/EtOAc (n = 3). Only technical replicate measurements we performed for E7 (n = 4).


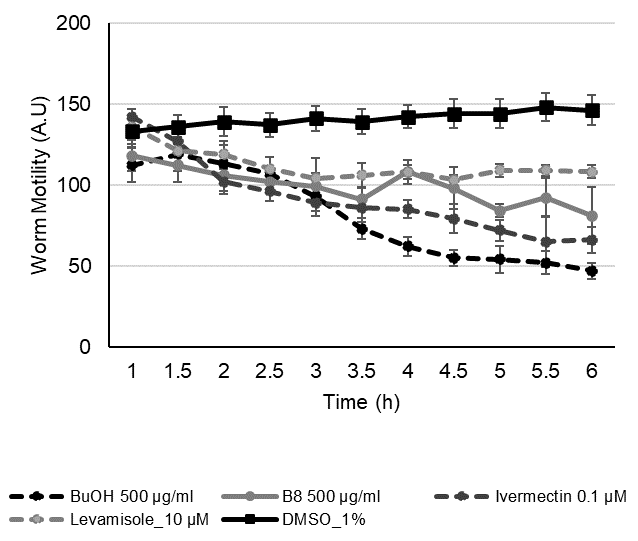


Figure S5: Concentration-response effect of B8 and BuOH Fraction (c = 150 µg/ml, technical replicates n =3) on worm motility compared to 10 µM levamisole and 0.1 µM ivermectin.

- - 1. **ED assay fractions**

**
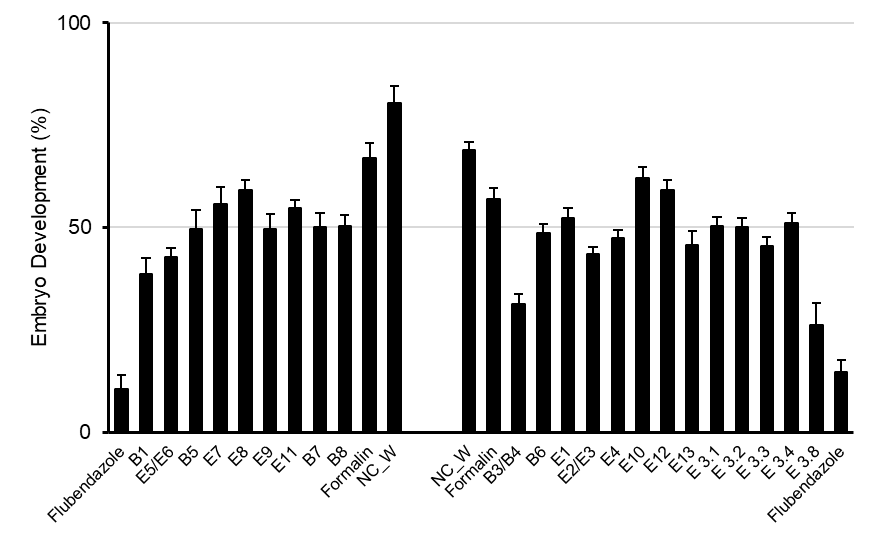
**

Figure S6: Mean percentage embryo development of A. galli eggs exposed to the C. alpina BuOH or EtOAc subfractions, negative controls NC_W (water), formalin, or positive control (flubendazole) over 28 days.

**
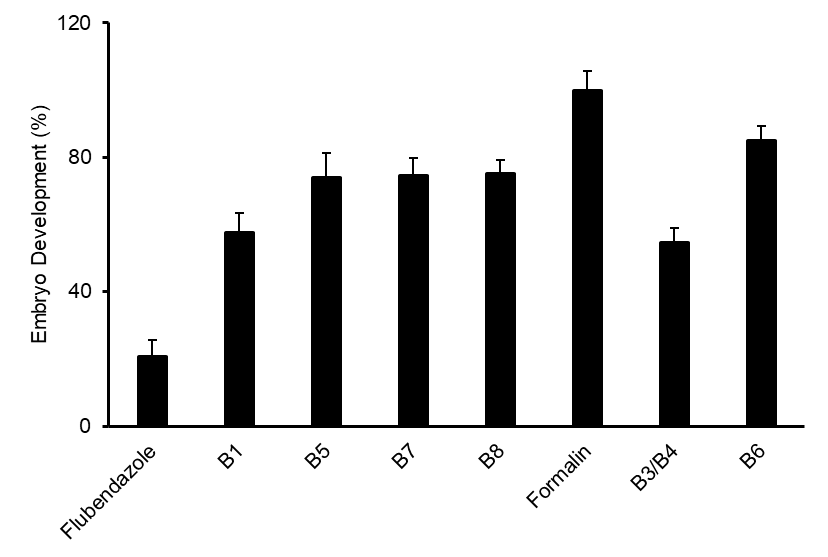
**

Figure S7: Percentage of developed A. galli embryos normalised to the formalin control (Mean ± SEM) during 28 days of exposure to the C. alpina BuOH subfractions, positive control (Flubendazole) and negative controls (Formalin).

**
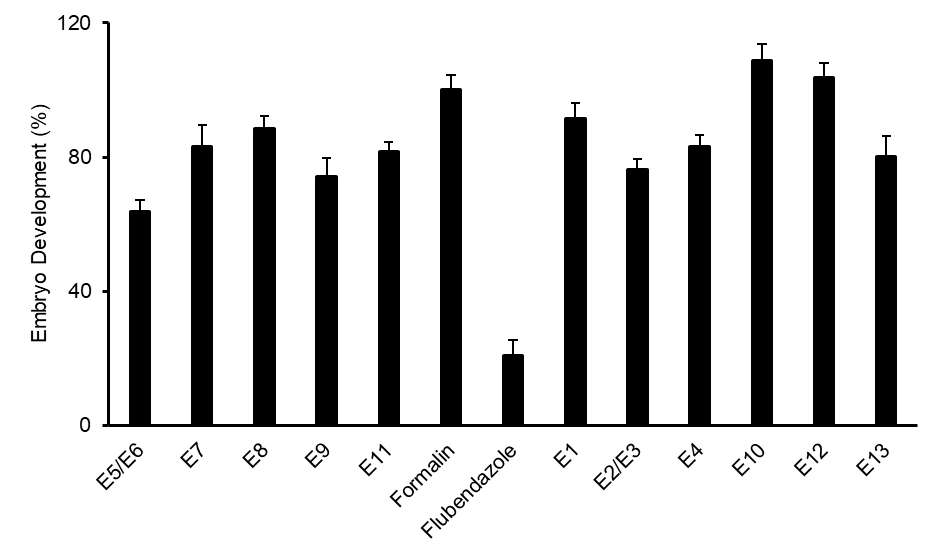
**

Figure S8: Percentage of developed A. galli embryos normalised to the negative control (Mean ± SEM) for 28 days exposure to the C. alpina EtOAc subfractions, positive control (Flubendazole) and negative controls (Formalin).

**
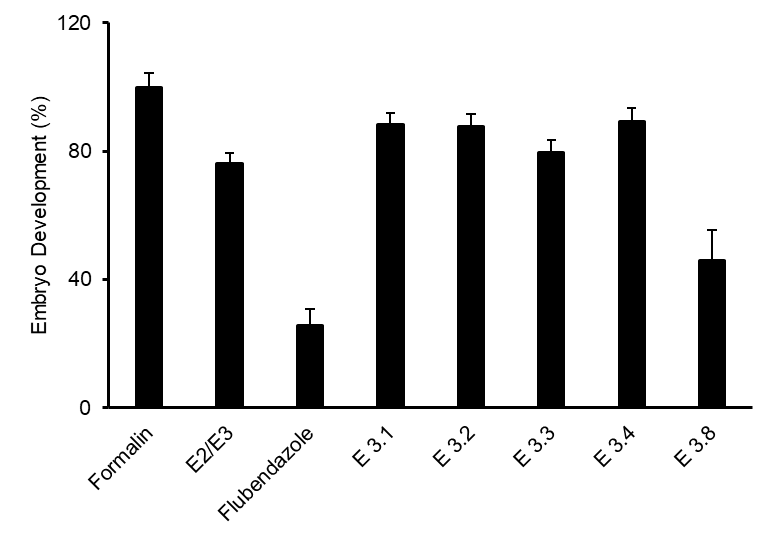
**

Figure S9: Percentage of developed A. galli embryos normalised to the negative control (Mean ± SEM) during 28 days exposure to A) E3 SEC derived subfractions, positive control (Flubendazole), and negative controls (Formalin).

**
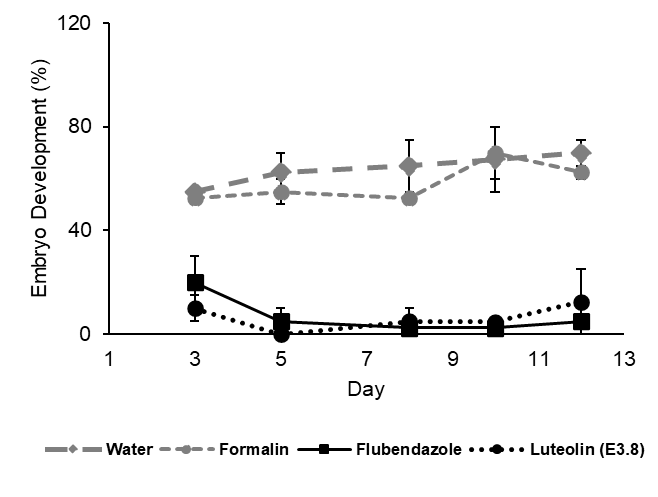
**

Figure S10: The effect of luteolin on A. galli embryo development (Mean ± SEM) over 12 days compared to the positive control (Flubendazole) and negative controls (Formalin, Water).

- - 1. **Tubulin assays**

**
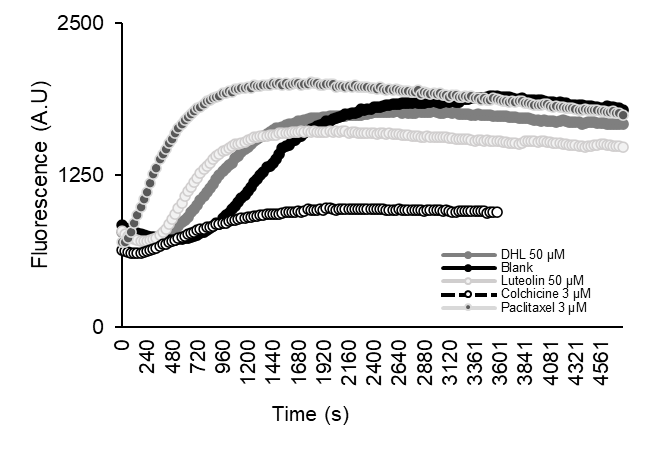
**

Figure S11: Tubulin polymerisation assay demonstrating the increase and decrease of fluorescence signal intensity of tubulin incubated with paclitaxel, colchicine, luteolin, and 11β,13-dihydrolactucin and compared to the blank.


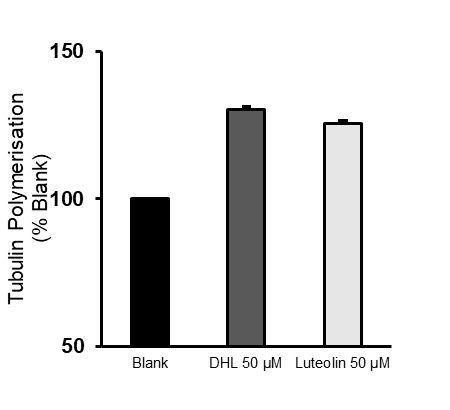


Figure S12: The tubulin polymerisation enhancing effect of 11β,13-dihydrolactucin and luteolin (c = 50 µM) compared to the blank control. Data shown as experimental technical replicates (n = 2) and expressed as % of blank (Mean ± SD).

- 1. **Phytochemical analysis**
     1. **HPLC-DAD-MS measurements**


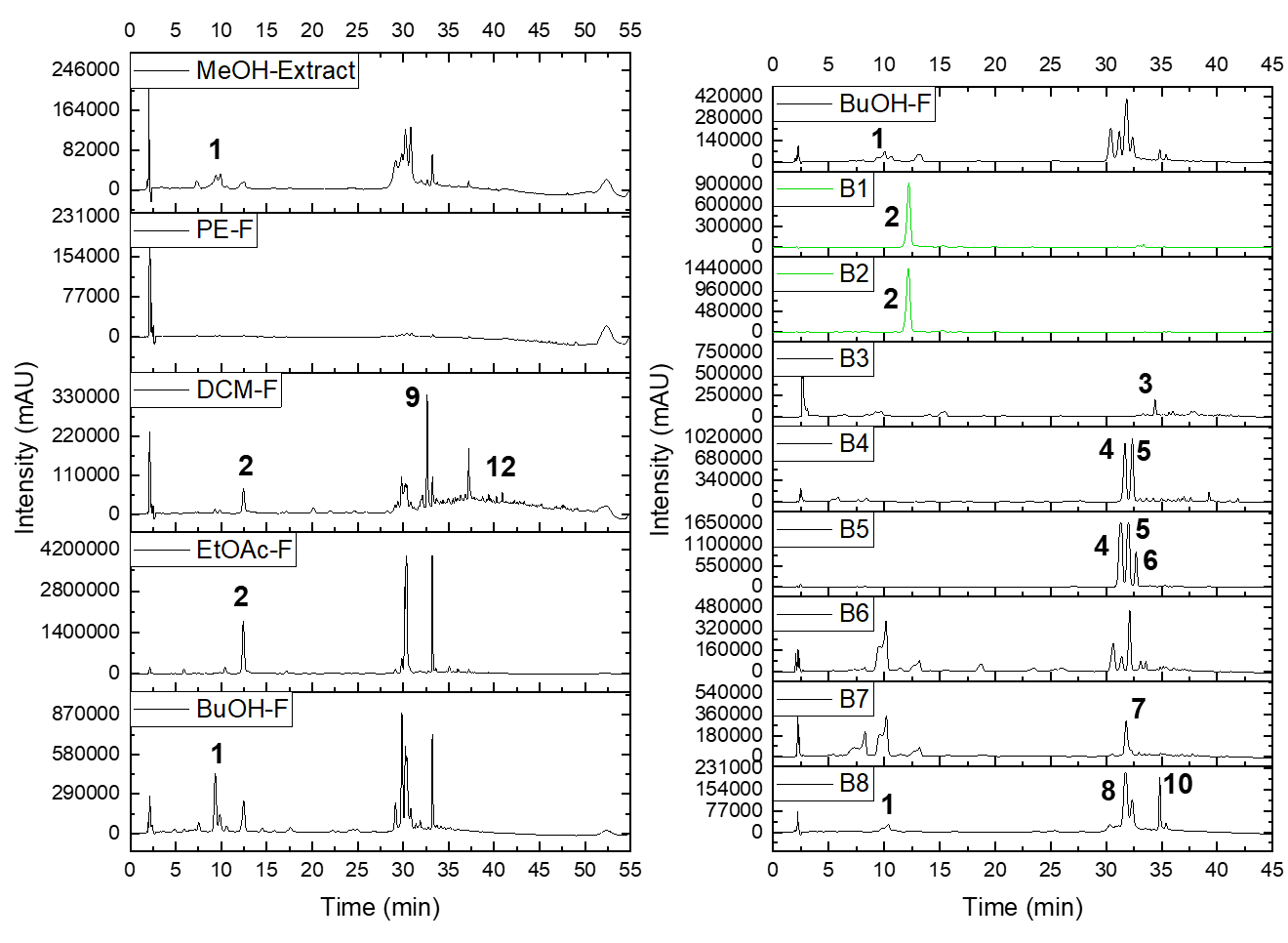


Figure S13 **:** HPLC chromatograms at 254 nm wavelength of the liquid-liquid fractions and FCC obtained BuOH subfractions labelled with peak assignments.


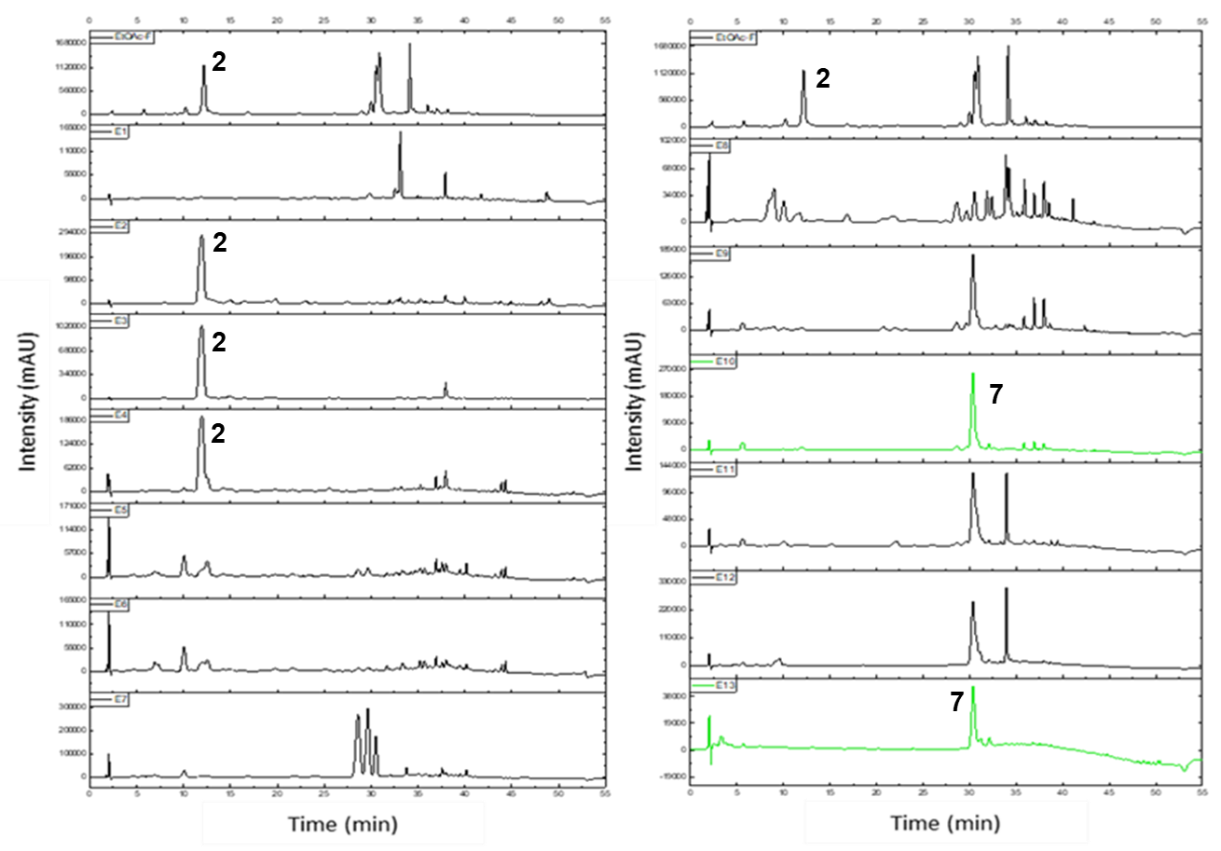


Figure S14**:** HPLC chromatograms at 254 nm wavelength of the FCC obtained EtOAc subfractions.


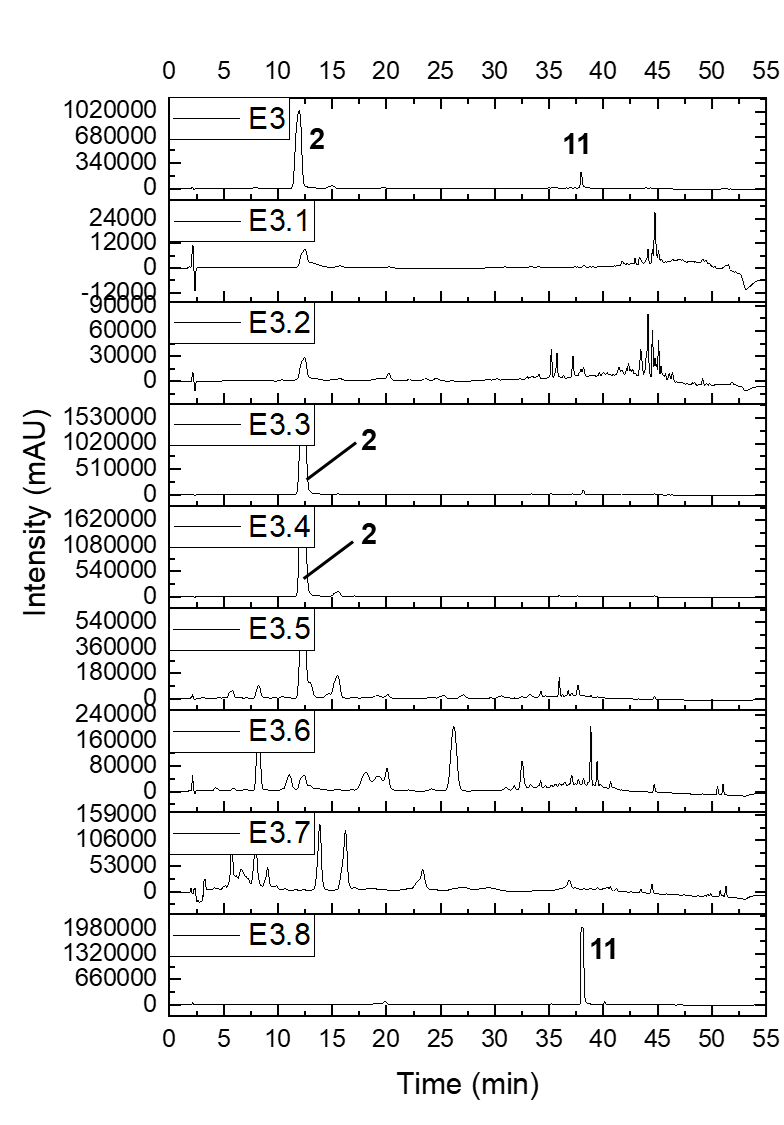


Figure S15: HPLC chromatograms at 254 nm wavelength of the FCC obtained EtOAc subfractions.

Table S1: Results from HPLC-UV analysis with tentative assignments.

| Peak | Rt (min) | UV_λmax_ (nm) | Reference  UV_λmax_ (nm) or Rt (min) | Internal  Standard | Assignment |
| --- | --- | --- | --- | --- | --- |
| 1 | 9.8 | 245, 300 (*sh*), 326 | 243, 325 **^(6)^** | Rt (min) = 9.8, UV_λmax_ (nm) = 234, 300 (*sh*), 331 | chlorogenic acid  (mono-O-caffeoylquinic acid) |
| 2 | 12.2 | 258  261 (B2) | - | *Rt(min) = 12.4, UV_λmax_ (nm) = 208, 259 | 11β,13-dihydrolactucin |
| 3 | 34.4 | 240, 270 | - | n.d. | Lactucin derivative |
| 4 | 31.7 | 257 (B4) , | 255 **^(7)^**  Rt = 30 min**^(8)^** | UV_λmax_ (nm) = 245, 269  Rt (min) = *28.5 | 8-acetyl-15β-D -glucopyranosyllactucin |
| 5 | 32.3 | 257 | Elutes at later Rt than **5^(9)^** | UV_λmax_ (nm) = 249, 269  Rt (min) = *29.6 | 8-acetyl-glucopyranosyllactucin  derivative**^(9)^** |
| 6 | 32.7 | 262 | Derivatives elute at later R_t_ than **2**^9^ | UV_λmax_ (nm): 208 and 259 | lactucin derivative |
| 7 | 31.7 | 254-255, 268 (*sh*), 348 | 255, 269 (*sh*), 349 **^(10)^**  Rt = 28-31 min**^(11)^** | Luteolin = UV_λmax_ (nm) = 254, 268 (*sh*), 349 | Flavonoid derivative/glycoside  (also strong absorbance signal at 366 nm) |
| 8 | 31.6 | 247, 300 (*sh*), 329 | 248, 300, 329 **^(12)^** | Rt (min) = *30.9, UV_λmax_ (nm) =244, 300 (*sh*), 327 | 3,5-dicaffeyolquinic acid |
| 9 | 34.6 | 257 | n.d. | n.d. | 8-deoxylactucin |
| 10 | 34.8 | 247, 300 (*sh*), 328 | 247, 300, 326  Elutes at later Rt than 8**^(12)^** | UV_λmax_ (nm) = 244, 300 (*sh*), 330 | 4,5-dicaffeyolquinic acid  (di-O-caffeoylquinic acid) |
| 11 | 38.6 | 254-255, 268 (sh), 348 |  | 254, 268 (*sh*), 349 | Luteolin |
| 12 | 44 |  |  |  | ostruthol |
| * = shift in retention time, the same as sample under repeat measurement.  (*sh*) = shoulder | | | | | |

Table S2: Results from MS analysis with tentative assignments.

| Peak | Rt (min) | [M+H]^+^  (m/z) | [M-H]^-^  (m/z) | Reference  Mass | Internal  Standard  (analysis) | Assignment |
| --- | --- | --- | --- | --- | --- | --- |
| 1 | 9.8 | 355.15 | 353 | [M−H]^−^= 353 m/z **^(8)^**  [M−H]^−^= 352.8 m/z**^(13)^** | n.d. | chlorogenic acid |
| 2 | 12.2 | 279.10 | 277 | [M+H]^+^ = 279 m/z**^(14)^** | [M+H]^+^ 279.12 m/z | 11β,13-dihydrolactucin |
| 7 | 31.7 | 449.1, 287.1 | 446.9, 285 | [M-H]^-^ = 446.7 m/z^13^ | n.d. | luteolin glycoside |
| 8 | 31.6 | 517.15 | n.d. | [M−H]^-^ = 515 m/z ^12^  [M−H]^-^ = 514.7 m/z^13^ | n.d. | dicaffeyolquinic acid derivative |
| 9 | 34.6 | 261.12 |  | [M+H]^+^ = m/z | n.d. | 8-deoxylactucin |
| 10 | 34.8 | 517.17 | 514.95 | [M−H]^-^ = 515 m/z ^12^  [M−H]^-^ = 514.7 m/z^13^ | n.d. | dicaffeyolquinic acid derivative |
| 11 | 38.6 | 287.1 | 285.1 | [M+H]^+^ = 287 m/z^10^ | n.d. | Luteolin |
| 12 | 44 | 387.3 | n.d. | [M+H]^+^ = 387.1 m/z^9^ | n.d. | ostruthol^7^ |

- - 1. **Structural elucidation of isolated compounds**

11β,13-dihydrolactucin

^1^H NMR (400 MHz, Methanol*-d_4_*): *δ*: 6.39 (dd, J = 2.6, 1.6 Hz, 1H, C*H* (3)), 4.82 (dd, J = 20.0, 1.9 Hz, 1H , C*H*_a_ (15)), 4.39 (dd, J = 18.7, 1.8 Hz, 1H, C*H*_b_ (15)), 3.70 (m, 2H, CH (5) + CH (8)), 3.68 – 3.62 (m, 1H, C*H* (6)), 2.82 (dd, J = 13.8, 10.7 Hz, 1H, C*H*_ax_ (9)), 2.65 (dq, J = 11.8, 6.9 Hz, 1H, C*H* (11)), 2.42 (s, 3H, C*H*_3_ (14)), 2.39 (dd, J = 13.7, 2.1 Hz, 1H, C*H*_eq_ (9)), 2.17 (dddd, J = 11.6, 9.7, 7.7, 1.5 Hz, 1H, C*H* (7)), 1.38 (d, J = 7.0 Hz, 3H, C*H*_3_ (13)) ppm. ^13^C-NMR (100 MHz, MeOD): δ 195.2, 177.9, 175.1, 147.3, 133.3, 132.9, 81.6, 69.8, 62.9, 61.9, 49.5, 49.4, 41.9, 21.3, 15.8 ppm. LCMS calcd for [C_15_H_16_O_5_]·H^+^ [M + H]^+^ = 279.1227; found, 279.7. [α]_D_^25^ (c = 0.5, CHCl_3_) = +8.3. UV (DMSO) λ_max_: 208 and 259 nm.

Luteolin

^1^H NMR (400 MHz, Methanol-*d*_4_) δ 7.41 (dd, *J* = 8.3, 2.3 Hz, 1H, C*H*(6‘)), 7.40 (brs, 1H, C*H*(2‘)) 6.92 (dd, *J* = 8.7, 0.9 Hz, 1H, C*H*(5‘)), 6.56 (s, 1H, C*H*(3)), 6.45 (d, *J* = 2.1 Hz, 1H, C*H*(8)), 6.22 (d, *J* = 2.1 Hz, 1H, C*H*(6)) ppm.

1. **References**

(1) Lionaki, E.; Tavernarakis, N. High-throughput and longitudinal analysis of aging and senescent decline in Caenorhabditis elegans. *Methods in molecular biology (Clifton, N.J.)* **2013**, *965*, 485-500. DOI: 10.1007/978-1-62703-239-1_32 From NLM. Porta-de-la-Riva, M.; Fontrodona, L.; Villanueva, A.; Cerón, J. Basic Caenorhabditis elegans methods: synchronization and observation. *J Vis Exp* **2012**, (64), e4019-e4019. DOI: 10.3791/4019 PubMed.

(2) Tiersch, K. M.; Daş, G.; Samson-Himmelstjerna, G. v.; Gauly, M. The role of culture media on embryonation and subsequent infectivity of Capillaria obsignata eggs. *Parasitology Research* **2013**, *112* (1), 357-364. DOI: 10.1007/s00436-012-3143-z.

(3) Rahimian, S.; Gauly, M.; Daş, G. Embryonation ability of Ascaridia galli eggs isolated from worm uteri or host faeces. *Veterinary Parasitology* **2016**, *215*, 29-34. DOI: <https://doi.org/10.1016/j.vetpar.2015.10.026>.

(4) von Samson-Himmelstjerna, G.; von Witzendorff, C.; Sievers, G.; Schnieder, T. Comparative use of faecal egg count reduction test, egg hatch assay and beta-tubulin codon 200 genotyping in small strongyles (cyathostominae) before and after benzimidazole treatment. *Veterinary Parasitology* **2002**, *108* (3), 227-235. DOI: <https://doi.org/10.1016/S0304-4017(02)00197-8>.

(5) Coles, G. C.; Jackson, F.; Pomroy, W. E.; Prichard, R. K.; von Samson-Himmelstjerna, G.; Silvestre, A.; Taylor, M. A.; Vercruysse, J. The detection of anthelmintic resistance in nematodes of veterinary importance. *Veterinary Parasitology* **2006**, *136* (3), 167-185. DOI: <https://doi.org/10.1016/j.vetpar.2005.11.019>.

(6) Robbins, R. J.; Bean, S. R. Development of a quantitative high-performance liquid chromatography–photodiode array detection measurement system for phenolic acids. *Journal of Chromatography A* **2004**, *1038* (1), 97-105. DOI: <https://doi.org/10.1016/j.chroma.2004.03.009>.

(7) Appendino, G.; Tettamanzi, P.; Gariboldi, P. Sesquiterpene lactones and furanocoumarins from Cicerbita alpina. *Phytochemistry* **1991**, *30* (4), 1319-1320. DOI: <https://doi.org/10.1016/S0031-9422(00)95225-7>.

(8) Fusani, P.; Zidorn, C. Phenolics and a sesquiterpene lactone in the edible shoots of Cicerbita alpina (L.) Wallroth. *Journal of Food Composition and Analysis* **2010**, *23* (6), 658-663. DOI: <https://doi.org/10.1016/j.jfca.2009.08.014>.

(9) Zheleva-Dimitrova, D.; Petrova, A.; Zengin, G.; Sinan, K. I.; Balabanova, V.; Joubert, O.; Zidorn, C.; Voynikov, Y.; Simeonova, R.; Gevrenova, R. Metabolite profiling and bioactivity of Cicerbita alpina (L.) Wallr. (Asteraceae, Cichorieae). *Plants* **2023**, *12* (5), 1009.

(10) Waridel, P.; Wolfender, J.-L.; Lachavanne, J.-B.; Hostettmann, K. Identification of the polar constituents of Potamogeton species by HPLC-UV with post-column derivatization, HPLC-MSn and HPLC-NMR, and isolation of a new ent-labdane diglycoside. *Phytochemistry* **2004**, *65* (16), 2401-2410. DOI: <https://doi.org/10.1016/j.phytochem.2004.06.031>.

(11) Alexandru, L.; Pizzale, L.; Conte, L.; Barge, A.; Cravotto, G. Microwave-assisted extraction of edible Cicerbita alpina shoots and its LC-MS phenolic profile. *Journal of the Science of Food and Agriculture* **2013**, *93* (11), 2676-2682. DOI: <https://doi.org/10.1002/jsfa.6082> (acccessed 2024/03/11).

(12) Gouveia, S. C.; Castilho, P. C. Characterization of phenolic compounds in Helichrysum melaleucum by high-performance liquid chromatography with on-line ultraviolet and mass spectrometry detection. *Rapid Communications in Mass Spectrometry* **2010**, *24* (13), 1851-1868. DOI: <https://doi.org/10.1002/rcm.4585>.

(13) Poulopoulou, I.; Horgan, M. J.; Siewert, B.; Siller, M.; Palmieri, L.; Martinidou, E.; Martens, S.; Fusani, P.; Temml, V.; Stuppner, H.; et al. In vitro evaluation of the effects of methanolic plant extracts on the embryonation rate of Ascaridia galli eggs. *Veterinary Research Communications* **2022**. DOI: 10.1007/s11259-022-09958-9.

(14) Graziani, G.; Ferracane, R.; Sambo, P.; Santagata, S.; Nicoletto, C.; Fogliano, V. Profiling chicory sesquiterpene lactones by high resolution mass spectrometry. *Food Research International* **2015**, *67*, 193-198. DOI: <https://doi.org/10.1016/j.foodres.2014.11.021>.
